# OT-CATH, a conserved antimicrobial peptide in *Otariidae* species, exhibits potent activity against gram-negative bacteria

**DOI:** 10.1128/aac.01026-26

**Published:** 2026-10-01

**Authors:** Yixiao Song, Yufeng Ren, Juan Wang, Zifan Ye, Chenchen Shi, Xiangjin Kong, Xudong Jiao, Jingjing Zhang, Yipeng Wang

**Affiliations:** 1 Yantai Institute of Coastal Zone Research, Chinese Academy of Sciences 74620, Yantai, Shandong, China; 2 University of Chinese Academy of Sciences 74519, Beijing, China; 3 Research Institute of Marine Traditional Chinese Medicine (Qingdao Academy of Chinese Medical Sciences), Shandong University of Traditional Chinese Medicine 612367, Qingdao, China; 4 Department of Biopharmaceutical Sciences, College of Pharmaceutical Sciences, Soochow University 625752, Suzhou, Jiangsu, China; Shionogi Inc., Florham Park, New Jersey, USA

**Keywords:** OT-CATH, antimicrobial peptides, gram-negative bacteria, ESBL-producing *E. coli*, multiple mechanisms of action

## Abstract

The rapid emergence of multidrug-resistant gram-negative bacteria necessitates the development of effective antimicrobial agents to combat antimicrobial resistance. Here, we identified and characterized OT-CATH, a conserved cathelicidin-family antimicrobial peptide derived from *Otariidae* species. OT-CATH exhibited potent bactericidal activity against gram-negative bacteria, particularly extended-spectrum β-lactamase-producing *Escherichia coli*, with rapid killing kinetics, potent antibiofilm activity, and no detectable resistance after 40 serial passages. Mechanistically, OT-CATH bound lipopolysaccharide, disrupted both the outer and inner bacterial membranes, and translocated into the cytoplasm to interact with bacterial DNA. Membrane disruption further triggered intracellular reactive oxygen species accumulation, proton motive force dissipation, ATP depletion, and metabolic collapse, collectively leading to bacterial death. OT-CATH also demonstrated favorable stability, low cytotoxicity, and favorable pharmacokinetic properties with measurable pulmonary distribution. In a murine pneumonia model, OT-CATH significantly improved survival, reduced bacterial burdens in the lungs and bronchoalveolar lavage fluid, and alleviated lung injury. These findings identify OT-CATH as a promising membrane-targeting antimicrobial peptide for the treatment of multidrug-resistant gram-negative bacterial infections.

## INTRODUCTION

The crisis of bacterial resistance, particularly that mediated by gram-negative bacteria, is rapidly eroding the efficacy of existing anti-infective therapies (1). Clinical isolates typified by *Escherichia coli* producing extended-spectrum β-lactamase (ESBL) have developed extensive resistance to multiple classes of β-lactam antibiotics, including cephalosporins, rendering first-line treatments increasingly ineffective (2, 3). These pathogens can also develop resistance through classic mechanisms, including target mutations, horizontal gene transfer, and enhanced efflux pump systems (4, 5). In addition, they can further enter biofilm or bacterial persister states, thereby evading immune clearance and antibiotic killing at the metabolic level (6–8). Traditional small-molecule antibiotics based on the “single target–high-affinity binding” model have gradually become ineffective against such multilayered defense systems (9–11). Consequently, there is an urgent need to develop novel antibacterial agents that can simultaneously breach membrane barriers, interfere with energy metabolism, and reduce the likelihood of resistance evolution.

Antimicrobial peptides (AMPs), as core effector molecules of the innate immune system, are regarded as potential candidates to replace traditional antibiotics (12, 13). Unlike small-molecule antibiotics, AMPs typically bind preferentially to negatively charged bacterial membranes via electrostatic interactions and exert bactericidal effects synergistically through membrane disruption and interference with multiple intracellular targets (14, 15). This “multi-target synergistic” mode significantly reduces the likelihood of drug-resistant mutations (16, 17). Among numerous AMP families, cathelicidins have attracted considerable attention due to their wide distribution in vertebrates and remarkable structural diversity (18). Cathelicidins from different species or even within the same species exhibit substantial differences in amino acid sequence, secondary structure, and physicochemical properties, endowing them with diverse antibacterial mechanisms and broad biological functions (19, 20). To date, several cathelicidin-derived peptides from different sources have entered clinical research, demonstrating promising application prospects (21). Although numerous cathelicidins have demonstrated potent antimicrobial activity, their clinical translation remains limited by several challenges, including reduced activity under physiological conditions, insufficient stability, and host toxicity (22). In addition, it is noteworthy that most existing AMPs, including cathelicidins, display broad-spectrum bactericidal activity and lack selective targeting capability against gram-negative bacteria, which limits their application value in precise anti-infective therapy (23).

Nowadays, most studies and drug development on AMPs focus on terrestrial animals such as humans (LL-37) (17), cattle (Bac5 and Bac7) (24), and goats (OaBac5α and ChBac5) (25). Marine environments are rich in gram-negative pathogens and exert unique environmental pressures (26). These pressures have driven the adaptive evolution of immune defense systems in marine animals, supporting the notion that they harbor diverse antimicrobial molecules with strong environmental adaptability. To date, the diversity, evolutionary conservation, and antimicrobial potential of cathelicidins from marine animals remain largely underexplored. Previously, we systematically identified cathelicidins from marine animals and unexpectedly found that cathelicidins from *Otariidae* species, including *Callorhinus ursinus*, *Zalophus californianus*, and *Eumetopias jubatus*, are highly structurally conserved, with identical amino acid sequences in their mature peptides. This phenomenon has not been reported in previous studies, suggesting that this peptide may have arisen from convergent evolution in the *Otariidae* family to adapt to the marine environment and may possess potent antibacterial activity.

In this study, we identified and systematically characterized a highly conserved cathelicidin, designated OT-CATH, from members of the *Otariidae* family. Based on its amphipathic α-helical structure, strong cationic charge, and moderate hydrophobicity, OT-CATH represents a promising candidate for further investigation as a membrane-active AMP against multidrug-resistant (MDR) gram-negative bacteria. We evaluated its antimicrobial activity against clinically relevant multidrug-resistant gram-negative pathogens and investigated its effects on biofilms, bacterial persisters, and resistance development. Furthermore, the mode of action of OT-CATH was investigated. Finally, the therapeutic potential of OT-CATH was evaluated in a murine pneumonia model.

## RESULTS

### Identification, structural prediction, and phylogenetic analysis of OT-CATH

**Fig 1:**
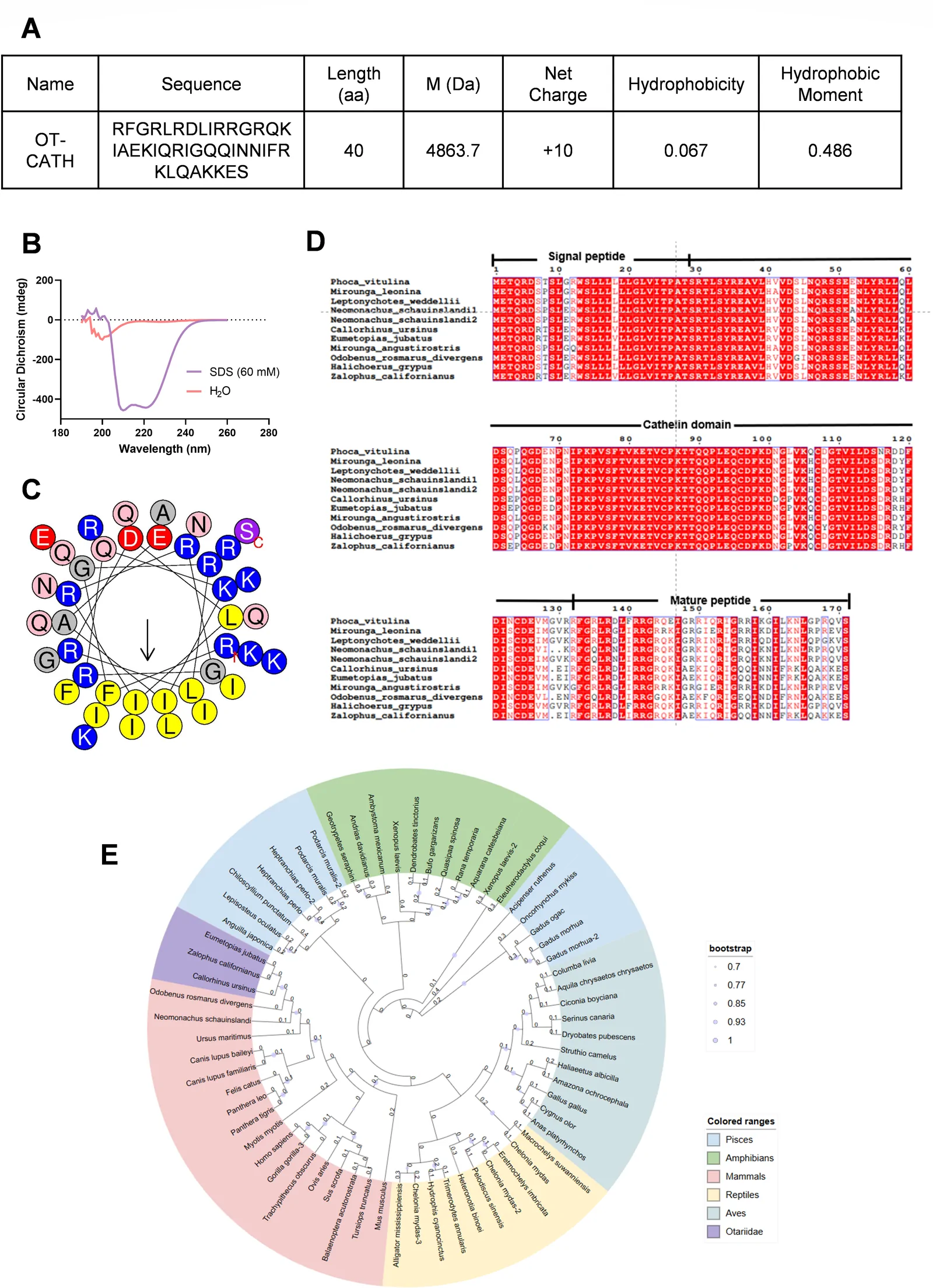
Sequence characteristics, secondary structure, and evolutionary analysis of OT-CATH. (**A**) Amino acid sequence and physicochemical properties (length, molecular weight, net charge, hydrophobicity, and hydrophobic moment) of the mature peptide. (**B**) Circular dichroism spectra of OT-CATH recorded in water and 60 mM SDS. (**C**) Helical wheel projection of the mature OT-CATH peptide, illustrating its amphipathic distribution. (**D**) Multiple sequence alignment of cathelicidin precursor proteins from representative *Otariidae* species. Conserved residues are highlighted in red, and the signal peptide, cathelin domain, and mature peptide are indicated above the alignment. (**E**) Phylogenetic analysis of representative vertebrate cathelicidin precursor proteins constructed using the neighbor-joining method with 1,000 bootstrap replicates. Branch support is indicated by blue circles, and branch lengths represent evolutionary distance.

Cathelicidin-related protein precursors typically consist of a highly conserved N-terminal signal peptide, a cathelin domain, and a hypervariable C-terminal mature peptide region. The strong conservation of the cathelin domain provides a reliable basis for cross-species identification of novel cathelicidins (27). Through systematic screening and BLAST alignment against the NCBI protein database of *Otariidae* species, we identified a novel cathelicidin-like precursor gene and named its C-terminal mature peptide OT-CATH (sequence: RFGRLRDLIRRGRQKIAEKIQRIGQQINNIFRKLQAKKES) (Fig. 1A). Physicochemical analysis showed that OT-CATH is a 40-amino-acid peptide with a molecular weight of 4,863.7 Da, a net charge of +10, a GRAVY value of 0.067, and a hydrophobic moment of 0.486 (Fig. 1A). Circular dichroism spectroscopy showed that OT-CATH exhibited a predominantly random-coil conformation in aqueous solution but adopted a typical α-helical structure in membrane-mimicking sodium dodecyl sulfate (SDS) solution (Fig. 1B), consistent with the structural characteristics of many membrane-active cathelicidins. Helical wheel projection further demonstrated a clear amphipathic organization, in which hydrophobic residues were concentrated on one face of the helix and positively charged residues on the opposite face (Fig. 1C) (28).

To investigate the evolutionary conservation of OT-CATH, we performed sequence alignment and phylogenetic analyses. As shown in Fig. 1D, this sequence is identical among three species from distinct genera of the *Otariidae* family: the northern fur seal (*Callorhinus ursinus*, XP_025741460.1), the California sea lion (*Zalophus californianus*, XP_027441316.1), and the Steller sea lion (*Eumetopias jubatus*, XP_027980039.1), showing high cross-genus conservation. Pinniped cathelicidin precursor proteins possess three well-defined functional domains: an N-terminal signal peptide, a central cathelin domain, and a C-terminal mature AMP. The cathelin domain exhibits nearly identical amino acid sequences and serves as the taxonomic marker of this family. To further analyze its evolutionary characteristics, we extended the alignment to 10 representative species covering the three major families of Pinnipedia (*Otariidae*, *Phocidae*, and *Odobenidae*). We found that OT-CATH exhibited multiple amino acid substitutions and significant sequence divergence in *Phocidae* and *Odobenidae* (Fig. 1D). These results indicate that OT-CATH is not an ancestral trait shared by all pinnipeds, but a functional element retained under strong purifying selection within the *Otariidae* lineage. Neighbor-joining phylogenetic analysis clustered vertebrate cathelicidins into five major clades corresponding to mammals, birds, reptiles, amphibians, and fishes (Fig. 1E). Cathelicidins from *Otariidae* formed a highly supported monophyletic clade (bootstrap = 100%), consistent with their close evolutionary relationship. OT-CATH clustered with other marine mammalian cathelicidins within the mammalian lineage.

### OT-CATH exhibits potent and selective antimicrobial activity against gram-negative bacteria

To comprehensively evaluate the antibacterial activity of OT-CATH, antimicrobial susceptibility was determined against a panel of clinically relevant multidrug-resistant isolates together with reference strains using both broth microdilution and CLSI-standardized disk diffusion assays. To facilitate comparison with established membrane-targeting antimicrobial agents, LL-37 and polymyxin B were included as comparator controls throughout all susceptibility assays. In addition, the antimicrobial susceptibility profiles of all clinical isolates against conventional antibiotics were determined according to CLSI guidelines and are summarized in Tables S1 to S7, confirming their multidrug-resistant phenotypes.

**Fig 2:**
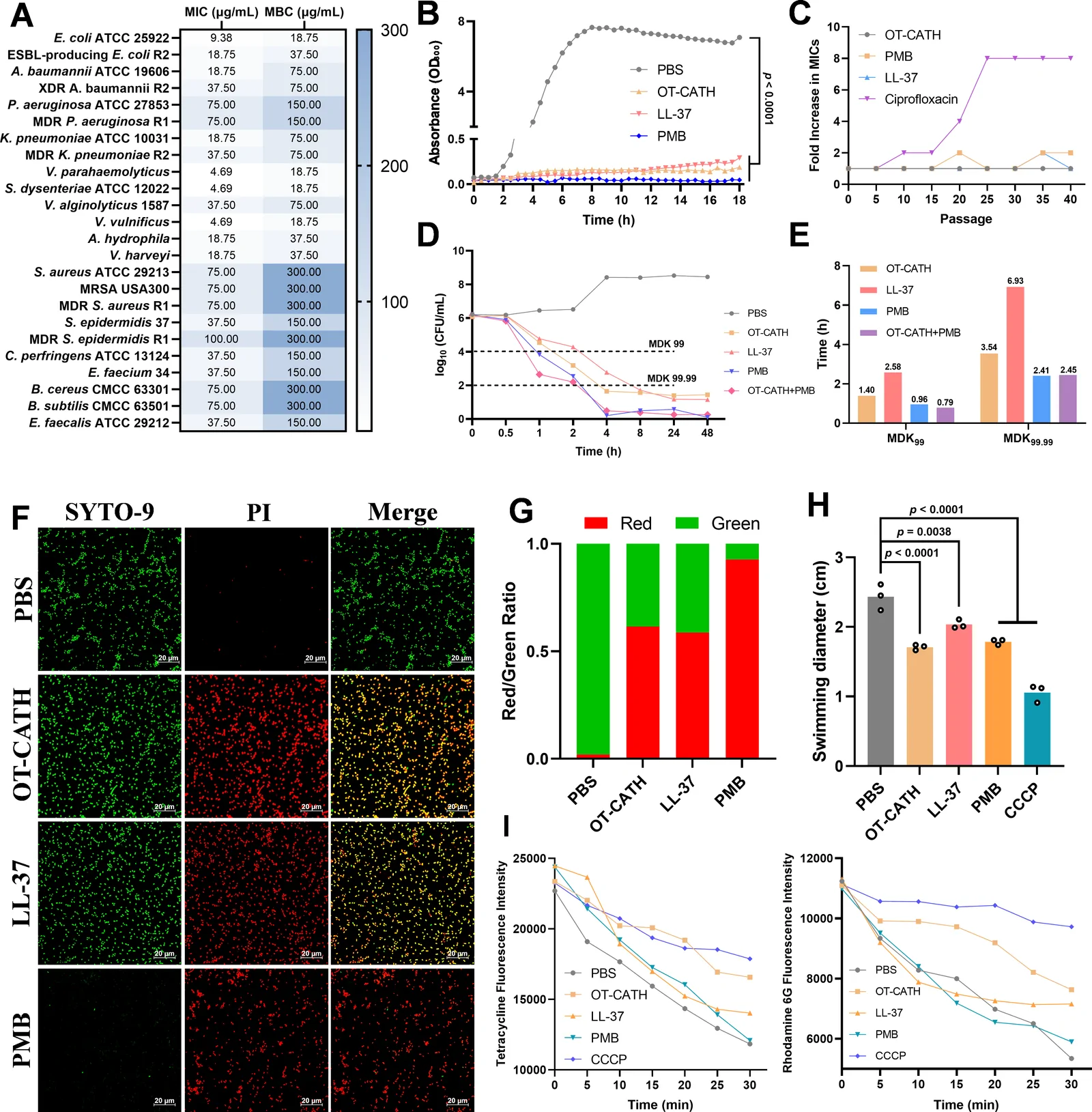
Antibacterial activity, bactericidal kinetics, resistance development, and membrane-associated effects of OT-CATH. (**A**) MIC (μg/mL) and MBC (μg/mL) of OT-CATH against gram-negative, gram-positive, and multidrug-resistant clinical isolates. (**B**) Bacterial growth curves monitored by OD_600_ following different peptide treatments. (**C**) Resistance induction during serial passaging in the presence of 1/2× MIC (MIC_OT-CATH_ = 18.75 µg/mL, MIC_LL-37_ = 75 µg/mL, MIC_PMB_ = 1.17 µg/mL, and MIC _Ciprofloxacin_ = 37.5 μg/mL) of the indicated agents. MIC values were determined every five passages. (**D**) Time-kill kinetics determined by CFU enumeration at the indicated time points. Dashed horizontal lines indicate the MDK_99_ and MDK_99.99_ bactericidal thresholds. (**E**) Time required to reach the MDK_99_ and MDK_99.99_ bactericidal endpoints under different peptide treatments. (**F**) Representative confocal laser scanning microscopy images of ESBL-producing *E. coli* R2 stained with SYTO 9 (green) and propidium iodide (PI, red) after 1 h at 5× MIC peptide treatment. Single SYTO 9, single PI, and merged fluorescence images are shown. Scale bar, 20 μm. (**G**) Quantification of the red-to-green fluorescence intensity ratio from SYTO 9/PI dual staining using ImageJ. (**H**) Swimming motility of ESBL-producing *E. coli* R2 on 0.3% agar after 16 h at 1/2× MIC peptide treatment. (**I**) Efflux pump activity of ESBL-producing *E. coli* R2 determined by tetracycline or rhodamine 6G at 5× MIC peptide treatment. Data are presented as mean ± SD from three independent biological experiments. Statistical significance is indicated in the figures, and each data point represents one independent biological replicate.

As shown in Fig. 2A, OT-CATH exhibited potent antibacterial activity against gram-negative bacteria, with MIC values ranging from 4.69 to 37.50 μg/mL (Table S3), whereas substantially higher MICs were required against most gram-positive bacteria (37.50–100 μg/mL) (Table S6). The representative ESBL-producing *E. coli* R2 strain showed a minimum inhibitory concentration (MIC) of 18.75 μg/mL. For some tested strains, the minimum bactericidal concentration (MBC)/MIC ratios were ≤4, indicating that OT-CATH exerts bactericidal rather than bacteriostatic activity (Fig. 2A). Consistent with the MIC results, disk diffusion assays demonstrated clear inhibition zones against gram-negative isolates but little or no activity against most gram-positive strains (Tables S4 and S7). LL-37 and polymyxin B were included as comparator membrane-targeting agents in all susceptibility assays, and the antimicrobial resistance genotypes and phenotypic susceptibility profiles of all clinical isolates are summarized in Table S1. Based on its well-characterized resistance profile, biofilm-forming ability, and reproducible persister formation, ESBL-producing *E. coli* R2 was selected for subsequent mechanistic studies (29).

Growth inhibition assays showed that OT-CATH effectively suppressed the proliferation of ESBL-producing *E. coli* R2 (Fig. 2B). At 5× MIC (93.75 μg/mL), bacterial growth was almost completely inhibited throughout the incubation period, exhibiting an inhibitory effect comparable to polymyxin B. To evaluate the propensity of OT-CATH to induce resistance, serial passaging was performed for 40 consecutive passages. As shown in Fig. 2C, repeated exposure to OT-CATH did not result in any detectable increase in MIC throughout the experiment. Similar results were obtained for LL-37 and polymyxin B. In contrast, ciprofloxacin progressively selected resistant populations, resulting in an approximately eightfold increase in MIC after serial passaging. These findings indicate that OT-CATH exhibits a low propensity to induce stable resistance during prolonged exposure. Time-kill assays further demonstrated the rapid bactericidal activity of OT-CATH against ESBL-producing *E. coli* R2 (Fig. 2D). Following treatment at 5× MIC, OT-CATH rapidly reduced the viable bacterial population, with bacterial counts approaching the limit of detection within 4 h. Compared with the two membrane-targeting comparators, OT-CATH exhibited faster killing kinetics than LL-37 but was slightly slower than polymyxin B. To further evaluate the ability of OT-CATH to eliminate tolerant bacterial subpopulations, the minimum duration required to kill 99% (MDK_99_) and 99.99% (MDK_99.99_) of the bacterial population was determined (Fig. 2E). OT-CATH exhibited shorter MDK_99_ and MDK_99.99_ values than LL-37, whereas both values remained slightly longer than those of polymyxin B. These results indicate that OT-CATH efficiently eliminates tolerant bacterial subpopulations and exhibits bactericidal activity intermediate between LL-37 and polymyxin B.

To assess whether OT-CATH affects bacterial membrane integrity, live/dead staining with SYTO 9 and propidium iodide (PI) was performed. Following treatment with OT-CATH at 5× MIC for 1 h, a marked increase in PI-positive fluorescence was observed compared with the PBS control, indicating substantial membrane permeabilization (Fig. 2F). Similar staining patterns were observed in both ESBL-producing *E. coli* R2 and *Acinetobacter baumannii* R2. Quantitative analysis of fluorescence intensity further confirmed significantly increased PI fluorescence after OT-CATH treatment, with membrane-disrupting activity greater than that of LL-37 but slightly weaker than polymyxin B (Fig. 2G).

### The inhibitory activity of OT-CATH against efflux pumps and bacterial motility

Because bacterial motility contributes to surface colonization and biofilm formation, swarming assays were subsequently performed on soft agar plates (0.3%). OT-CATH significantly reduced the swarming diameter of ESBL-producing *E. coli* R2 compared with the untreated control, exhibiting stronger inhibition than LL-37 and an effect comparable to polymyxin B (Fig. 2H). To further investigate whether OT-CATH influences bacterial efflux function, intracellular accumulation of tetracycline and rhodamine 6G was measured (30, 31). OT-CATH treatment significantly increased the intracellular accumulation of both tetracycline and rhodamine 6G, producing fluorescence curves similar to those of the positive control CCCP. CCCP is an oxidative phosphorylation uncoupler that dissipates the proton motive force (PMF). Given that PMF provides the driving force required for bacterial motility, OT-CATH may impair bacterial movement via PMF disruption. The inhibitory effect of OT-CATH was greater than that of LL-37 but slightly weaker than polymyxin B (Fig. 2I).

### Antibiotic synergy of OT-CATH

**Fig 3:**
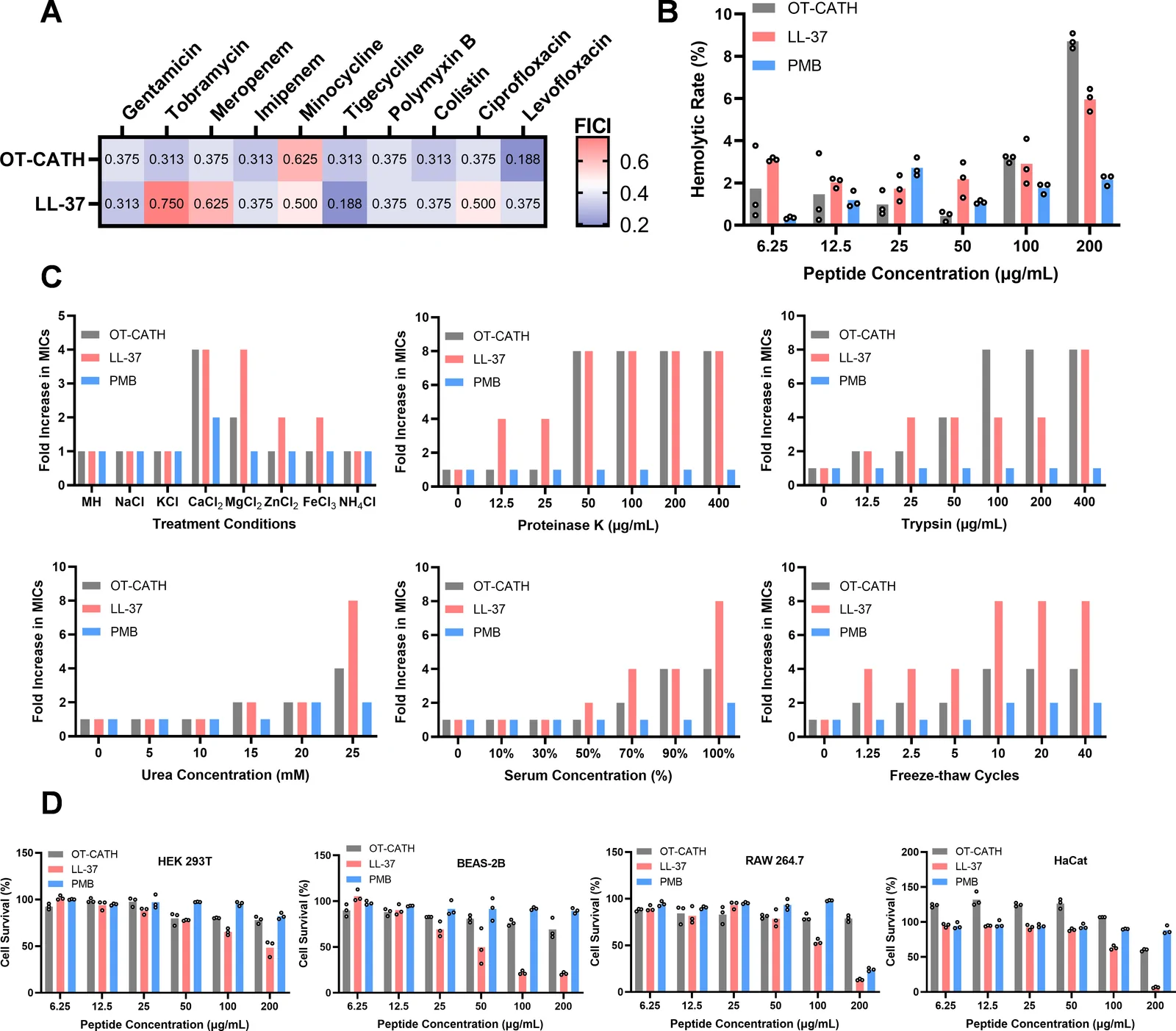
Synergistic activity, hemolysis, stability, and cytotoxicity of OT-CATH. (**A**) Fractional inhibitory concentration index of OT-CATH in combination with various clinically used antibiotics for synergy evaluation. LL-37 was included as an activity control. (**B**) Hemolytic activity of OT-CATH against mouse erythrocytes was assessed by measuring the supernatant absorbance at 540 nm after incubation with various peptide concentrations at 37°C for 30 min. Triton X-100 was used as the positive control (100% hemolysis), and PBS was used as the negative control. LL-37 and polymyxin B were included as activity controls. (**C**) Stability of OT-CATH under various physicochemical conditions. Metal ions were directly supplemented into MH medium. For other conditions (proteases, urea, and serum), the peptide was pre-incubated at 37°C for 2 h before the remaining antibacterial activity was assessed by MIC assay. LL-37 and polymyxin B were included as activity controls. (**D**) Cytotoxicity evaluation of OT-CATH on normal mammalian cells. Cell viability of mouse macrophage RAW264.7, human skin keratinocyte HaCaT, human embryonic kidney epithelial HEK293T, and human bronchial epithelial BEAS-2B cells was assessed by MTT assay after treatment with various concentrations of OT-CATH. LL-37 and polymyxin B were included as activity controls. All experiments were performed in three biological replicates, and data are presented as mean ± SD. One-way ANOVA was used for statistical analysis in panel **C**, with *P*-values indicated. Each data point represents an individual replicate.

AMPs have been widely shown to enhance antibiotic susceptibility in drug-resistant bacteria and reverse resistance (32, 33). To explore the combinational therapeutic potential of OT-CATH, 10 clinically common antibiotics covering aminoglycosides, carbapenems, tetracyclines, polymyxins, and quinolones were selected in this study. Using the classic AMP LL-37 as a control, the checkerboard dilution method was adopted to determine the fractional inhibitory concentration index (FICI) of the two peptides combined with antibiotics against ESBL-producing *E. coli* R2. As presented in Fig. 3A, OT-CATH exerted synergistic antibacterial effects with most tested antibiotics, with FICI values ranging from 0.188 to 0.625. Except for the additive effect observed in combination with minocycline (FICI = 0.625), the FICI values of all other combinations were lower than 0.5. The control peptide LL-37 exhibited synergism with some antibiotics; no synergistic activity was detected when combined with tobramycin and meropenem, with FICI values reaching up to 0.750 and 0.625, respectively. Overall, OT-CATH displayed a broader-spectrum and more stable *in vitro* synergistic antibacterial property compared with LL-37. Notably, OT-CATH reduced the MIC values of multiple antibiotics by four- to eightfold, indicating its promising prospect for clinical combined application.

### Stability and biosafety of OT-CATH

Ensuring the safety and stability of a drug is critical for its clinical application. We first evaluated the hemolytic activity of OT-CATH against mouse red blood cells, with LL-37 and PMB serving as controls for all biosafety experiments. As shown in Fig. 3B, the hemolysis rate of OT-CATH against mouse erythrocytes remained below 10% even at a high concentration (200 μg/mL). In comparison, LL-37 exhibited similarly low hemolysis, ranging from 2.0% to 6.5% across the tested concentrations, while PMB showed the lowest hemolytic activity, with values consistently below 2.2% at all concentrations. These results indicate that all three peptides possess favorable hemocompatibility, with OT-CATH showing a safety profile comparable to that of the well-studied LL-37.

In the stability assessment, we evaluated the stability of OT-CATH under different salt ion environments, repeated freeze–thaw cycles, protease treatments, urea exposure, and incubation with varying concentrations of mouse serum. LL-37 and PMB were included as parallel controls in all stability tests. All results are expressed as the fold increase in MICs, where higher values indicate greater loss of activity. As shown in Fig. 3C, OT-CATH exhibits favorable physicochemical and enzymatic stability under most physiologically relevant conditions. Its antibacterial activity remains largely unaffected by physiological concentrations of monovalent ions (Na^+^ and K^+^), repeated freeze–thaw cycles (up to 10 cycles), low concentrations of urea (≤15 mM), and different concentrations of mouse serum. Upon exposure to divalent cations (Mg²^+^ and Ca²^+^), moderate urea levels (10–40 mM), and proteolytic enzymes, the MIC values of the peptide generally increased by no more than fourfold. The slight reduction in activity observed following protease treatment is commonly seen among linear cationic peptides and does not impair its overall stability profile. These results support the potential of OT-CATH as a stable candidate suitable for further preclinical development.

Furthermore, we comprehensively evaluated the cellular cytotoxicity of OT-CATH. As shown in Fig. 3D, the MTT assay was utilized to assess cytotoxicity toward four mammalian cell lines: human embryonic kidney HEK 293T cells, human bronchial epithelial BEAS-2B cells, mouse macrophage RAW264.7 cells, and human immortalized keratinocyte HaCaT cells. For RAW264.7 cells, cell viability remained above 80% even at the highest tested concentration of 200 μg/mL, without obvious dose-dependent reduction. For HEK 293T and BEAS-2B cells, OT-CATH maintained cell viability above 70% across the entire concentration gradient up to 200 μg/mL. OT-CATH exerted concentration-dependent toxicity against HaCaT cells: cell viability was comparable to the untreated control at concentrations ranging from 6.25 to 100 μg/mL, whereas viability declined to approximately 60% at 200 μg/mL. By comparison, LL-37 exhibited evident cytotoxicity at elevated concentrations across all four cell lines. Overall, OT-CATH exhibits favorable biocompatibility toward multiple mammalian cell types at physiologically relevant concentrations, providing important toxicological evidence to support subsequent animal studies and formulation development.

### Anti-biofilm and anti-persister activity of OT-CATH

**Fig 4:**
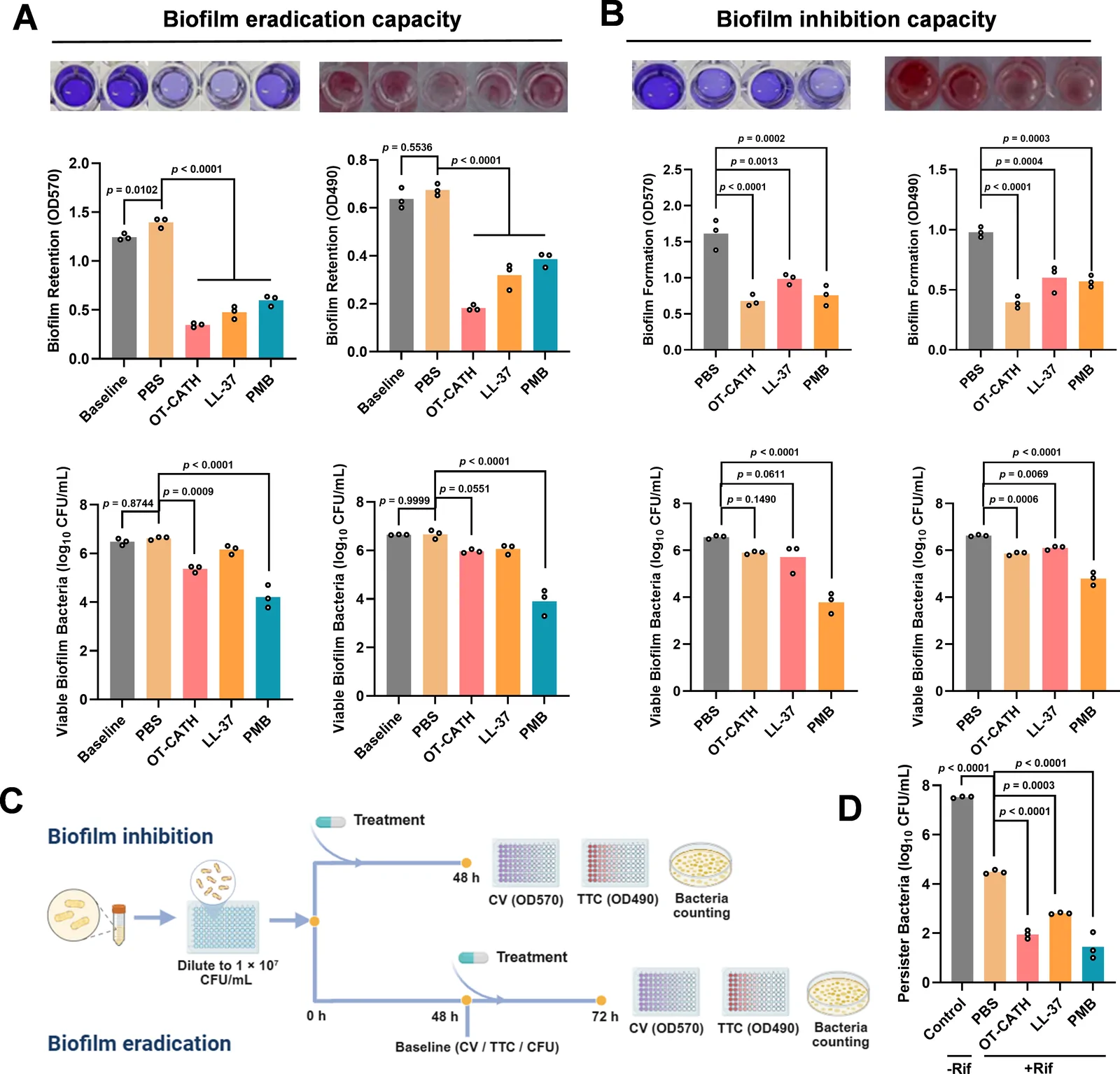
Antibiofilm and anti-persister efficacy of OT-CATH. (**A**) Biofilm eradication capacity. Pre-formed biofilms were established by incubating bacteria (1 × 10^7^ CFU/mL) for 48 h. The baseline biofilm biomass and viable cell counts were measured before treatment. Biofilms were then treated with PBS (control), OT-CATH, LL-37, or PMB for an additional 24 h. Biofilm biomass was quantified using crystal violet staining (OD_570_), and metabolic activity was assessed via TTC reduction assay (OD_490_). Viable bacterial counts within the biofilms were determined by CFU assays. (**B**) Biofilm inhibition capacity. Bacterial suspensions (1 × 10^7^ CFU/mL) were co-incubated with different treatments (PBS, OT-CATH, LL-37, or PMB) for 48 h to assess the ability to prevent biofilm formation. Biofilm formation was evaluated by crystal violet staining (OD_570_) and TTC reduction assay (OD_490_). The number of biofilm-associated bacteria was quantified by CFU counting. (**C**) Schematic representation of the experimental workflow. The diagram illustrates the protocols for biofilm inhibition and eradication assays. For the inhibition assay, bacteria are treated simultaneously with biofilm formation for 48 h. For the eradication assay, biofilms are allowed to form for 48 h (baseline) before being subjected to treatment for another 24 h (total 72 h). Endpoints include crystal violet staining, TTC metabolic assay, and bacterial counting. (**D**) Persister bacteria were enriched by 100× MIC (MIC_Rif_ = 4.69 µg/mL) rifampicin treatment (+Rif), while untreated bacteria without rifampicin (−Rif) were set as control. Enriched persisters were incubated with the indicated agents for 12 h, and viable persister loads were quantified as log_10_ CFU/mL to assess persister-eradicating activity. All experiments were performed in three biological replicates; data are presented as mean ± SD. One-way ANOVA was used for statistical analysis, with *P*-values indicated. Each data point represents an individual replicate.

Biofilm formation is one of the key processes enabling bacterial persistence and the development of drug resistance (34). Within biofilm structures, bacteria are embedded in an extracellular polymeric substance matrix that shields them from environmental stresses, impairs antimicrobial penetration, markedly enhances pathogenicity, and can elevate resistance by up to 1,000-fold (35, 36). We evaluated the ability of OT-CATH to inhibit biofilm formation and eradicate preformed mature biofilms using crystal violet staining and 2,3,5-triphenyltetrazolium chloride (TTC) reduction assays, respectively. Crystal violet staining reflects total biofilm biomass, while TTC reduction indicates the metabolic activity of viable bacteria within the biofilm. For the biofilm eradication assay, mature biofilms were established for 48 h before treatment, and baseline biofilm biomass, metabolic activity, and viable bacterial counts were determined immediately before peptide exposure. As shown in Fig. 4A, OT-CATH significantly reduced biofilm biomass, metabolic activity, and the number of viable biofilm-associated bacteria compared with the PBS control. Its antibiofilm activity was stronger than that of LL-37 but slightly weaker than that of polymyxin B. We next investigated the effect of OT-CATH on biofilm formation by adding the peptide during the initial stage of biofilm development. As shown in Fig. 4B, OT-CATH effectively prevented biofilm formation, resulting in marked reductions in biofilm biomass, metabolic activity, and viable bacterial counts compared with the PBS control. Similar to the biofilm eradication assay, OT-CATH exhibited stronger inhibitory activity than LL-37 but remained slightly less effective than polymyxin B. The experimental workflows for the biofilm inhibition and biofilm eradication assays are summarized in Fig. 4C.

Furthermore, biofilm formation induces bacteria to enter a dormant state, generating a large population of persister bacteria that typically tolerate high doses of antibiotics due to their low metabolic activity (37). These bacteria can survive exposure to rifampicin at 100× MIC. We further performed plate colony-counting assays following OT-CATH treatment. As shown in Fig. 4D, OT-CATH markedly decreased the surviving persister population compared with the PBS control. Its anti-persister efficacy was intermediate between those of LL-37 and polymyxin B. Collectively, these results demonstrate that OT-CATH effectively inhibits biofilm formation, reduces established biofilms, and eliminates antibiotic-tolerant bacterial persisters.

### OT-CATH disrupts bacterial membrane integrity

**Fig 5:**
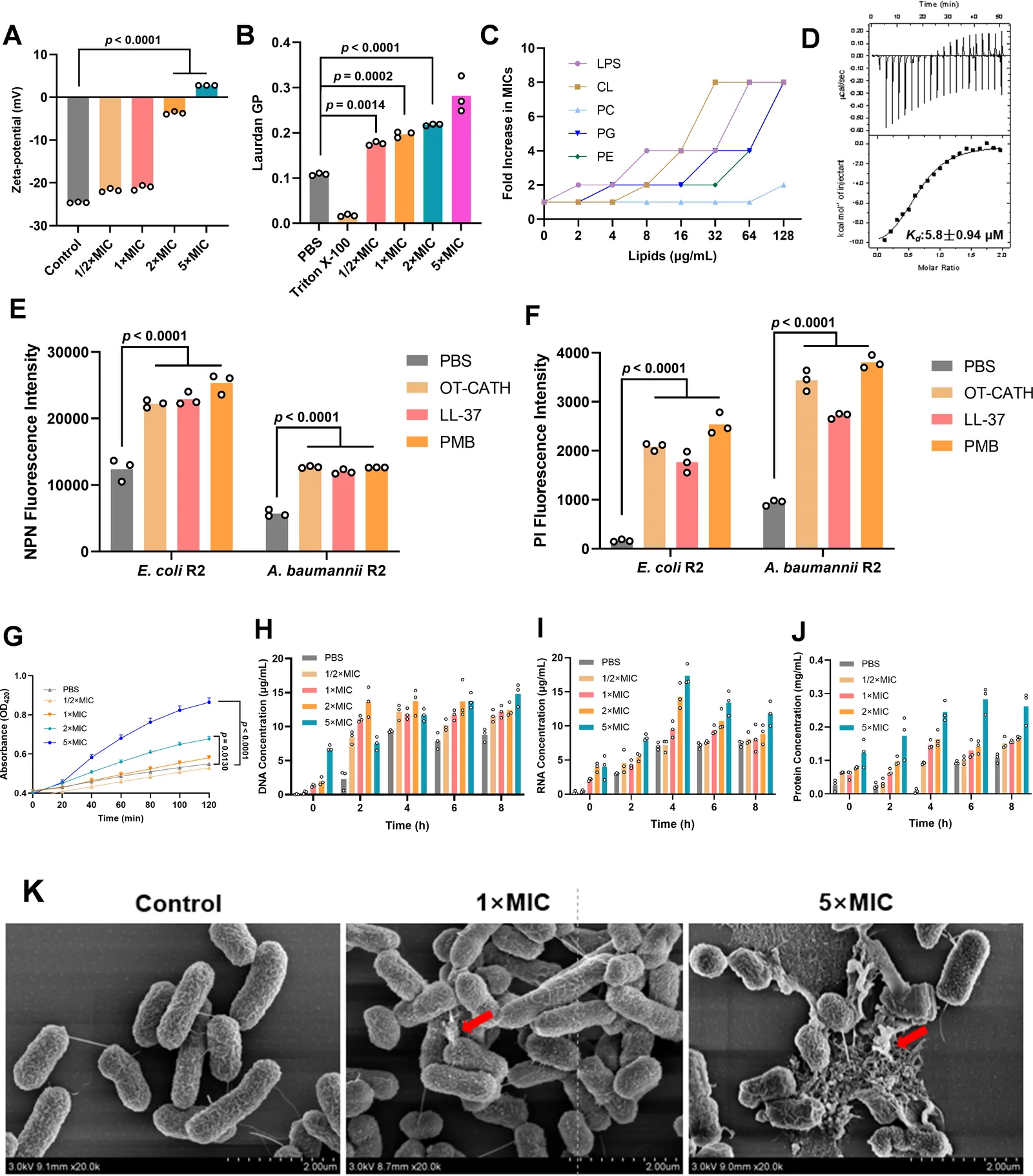
Membrane-disrupting effects of OT-CATH. (**A**) Zeta potential of ESBL-producing *E. coli* R2 after incubation with OT-CATH, indicating the degree of peptide accumulation on the membrane surface. (**B**) Membrane fluidity quantified by Laurdan GP. Fluorescence excitation was set at 350 nm, and emission signals were acquired at 440 and 490 nm. Measurements were conducted on bacteria following 30 min peptide treatment. (**C**) The exogenous lipid competition assay, the antibacterial activity of OT-CATH, determined by checkerboard microdilution assay with doses ranging from 0 to 128 μg/mL. (**D**) ITC analysis of the interaction between OT-CATH and LPS. Upper panel, raw thermograms. Lower panel, integrated binding isotherm fitted using a one-site binding model. (**E**) Outer membrane permeability of ESBL-producing *E. coli* R2 and *A. baumannii* R2 after 30 min of peptide treatment, determined by the 1-N-phenylnaphthylamine (NPN) uptake assay (excitation, 350 nm; emission, 420 nm). (**F**) Inner membrane permeability of ESBL-producing *E. coli* R2 and *A. baumannii* R2 after 30 min of peptide treatment, determined by PI uptake (excitation, 535 nm; emission, 615 nm). (**G–J**) Treatment with OT-CATH for 120 min induced the leakage of intracellular contents, including cytoplasmic β-galactosidase, DNA, RNA, and proteins. (**K**) Representative scanning electron microscopy images of ESBL-producing *E. coli* R2 after 1 h of treatment with OT-CATH at the indicated concentrations. Scale bars, 2 μm. All experiments were performed in three biological replicates; data are presented as mean ± SD. One-way ANOVA was used for statistical analysis, with *P*-values indicated, and each data point represents an individual replicate.

Given the influence of divalent cations on the activity of OT-CATH and the electrostatic basis of cationic AMPs binding to negatively charged groups on bacterial surfaces (38), we hypothesized that it may act by targeting bacterial membranes. We first measured changes in bacterial membrane potential upon addition of OT-CATH, which reflects the strength of electrostatic interactions between the peptide and negatively charged bacterial surface components such as lipopolysaccharide (LPS) or phospholipid headgroups. As shown in Fig. 5A, the positively charged peptide OT-CATH neutralized negative surface charges. At 5× MIC, the surface potential of ESBL-producing *E. coli* R2 increased from −24.6 to 2.78 mV, indicating extensive accumulation of OT-CATH on the bacterial membrane surface. Accumulation of membrane-active compounds typically causes drastic changes in membrane fluidity (39). We therefore used the membrane-sensitive dye Laurdan to assess membrane alterations in ESBL-producing *E. coli* R2 upon exposure to OT-CATH. As shown in Fig. 5B, OT-CATH reduced membrane fluidity in a concentration-dependent manner. The GP value (higher values indicate lower fluidity) decreased to approximately 0.15 at 1/2× MIC and further to about 0.28 at 5× MIC. This likely results from electrostatic anchoring of the peptide to the membrane surface, restricting lipid motion, while insertion of its amphipathic sequence induces ordered packing of membrane lipids. We further analyzed negatively charged membrane targets in ESBL-producing *E. coli* R2, including outer membrane LPS, inner membrane phosphatidylglycerol (PG), and cardiolipin (CL), all canonical components of bacterial membranes. Addition of negatively charged LPS and CL strongly impaired the antibacterial activity of OT-CATH (eightfold MIC increase) in a concentration-dependent manner (Fig. 5C), consistent with our earlier hypothesis. To characterize the interaction between OT-CATH and LPS, isothermal titration calorimetry (ITC) was performed. As shown in Fig. 5D, sequential injections of OT-CATH into the LPS solution produced pronounced downward heat peaks, indicating an exothermic binding process. The magnitude of heat release gradually decreased with increasing injections, suggesting progressive saturation of binding sites. Integration of the heat signals and nonlinear fitting of the binding isotherm yielded a dissociation constant (*K_d_*) of 5.8 ± 0.94 µM, indicating a moderate binding affinity between OT-CATH and LPS. In addition, OT-CATH also disrupts the bacterial cell wall (Fig. S4), contributing to cellular damage. The fluorescent probe 7-hydroxy-4-methylcoumarin-3-acetic acid hydrazide-conjugated D-alanine (HADA) is specifically incorporated into newly synthesized peptidoglycan strands, and its fluorescence intensity directly reflects the activity and integrity of cell wall synthesis. Quantitative analysis showed that the HADA fluorescence signal was significantly enhanced even at 1/2× MIC (*P* < 0.0001), with a clear concentration-dependent trend. These results indicate that OT-CATH not only targets the bacterial outer membrane but also penetrates into the peptidoglycan layer, causing loosening and even rupture of the cell wall structure, thereby weakening bacterial osmotic resistance.

Membrane anchoring by AMPs is a critical prerequisite for membrane damage. We further assessed outer membrane permeability using the hydrophobic probe 1-N-phenylnaphthylamine (NPN), which fluoresces only upon entering hydrophobic membrane environments. The inner membrane permeability was assessed using the nucleic acid probe propidium iodide, which enters membrane-damaged cells and binds DNA to emit fluorescence. As shown in Fig. 5E and F, OT-CATH caused severe damage to both membranes. OT-CATH significantly increased both NPN and PI fluorescence, indicating disruption of the outer and inner bacterial membranes, respectively. Similar results were observed in both ESBL-producing *E. coli* R2 and *A. baumannii* R2. Compared with LL-37, OT-CATH exhibited greater membrane-permeabilizing activity, whereas polymyxin B produced the strongest effect in both assays. Membrane rupture or increased permeability typically leads to leakage of intracellular contents such as enzymes, nucleic acids, and proteins (40). We therefore evaluated extracellular leakage of β-galactosidase using an O-nitrophenyl-β-D-galactopyranoside (ONPG) hydrolysis assay. As shown in Fig. 5G, the hydrolysis rate of extracellular ONPG increased significantly following OT-CATH treatment at concentrations above 2× MIC, indicating impaired membrane barrier function and leakage of intracellular enzymes. Measurements of extracellular nucleic acid and protein levels (Fig. 5H through J) revealed massive nucleic acid leakage within 2 h. In contrast to DNA, RNA levels decreased after 4 h, likely due to partial degradation, but remained much higher than baseline. A significant increase in protein content occurred within 4 h, slightly later than nucleic acid release, further confirming that the peptide disrupts bacterial membrane integrity. These findings were supported by scanning electron microscopy (SEM) observations (Fig. 5K). Untreated bacterial cells exhibited intact, smooth morphology. In contrast, peptide-treated bacteria displayed severe surface irregularities, including wrinkling, pitting, pore formation, and, in some cases, complete lysis with massive leakage of cellular contents.

In summary, OT-CATH anchors to the bacterial membrane surface, disrupts the integrity of both outer and inner membranes, triggers leakage of intracellular contents and structural collapse, thereby efficiently killing bacteria in a multi-targeted and irreversible manner.

### OT-CATH triggers a cascade of downstream cellular events following membrane disruption

Previous studies have shown that some AMPs can disrupt cell membranes or participate in transmembrane transport. Subsequently, they can bind to bacterial genomic DNA, interfere with cellular activities, and induce metabolic disorders (41). We therefore investigated the binding affinity of OT-CATH to *E. coli* genomic DNA. We found that at a concentration ratio (μg/mL) of 1:3, OT-CATH completely inhibited DNA migration in agarose gels, indicating a strong DNA-binding effect (Fig. S5). In the EB competitive binding assay, as the concentration of OT-CATH increased from 20 to 120 μg/mL, the fluorescence intensity of the EB-DNA complex decreased in a dose-dependent manner. This result suggests that OT-CATH competes with EB for DNA-binding sites, with affinity increasing at higher concentrations, further confirming its direct nucleic acid-binding ability (Fig. S6).

**Fig 6:**
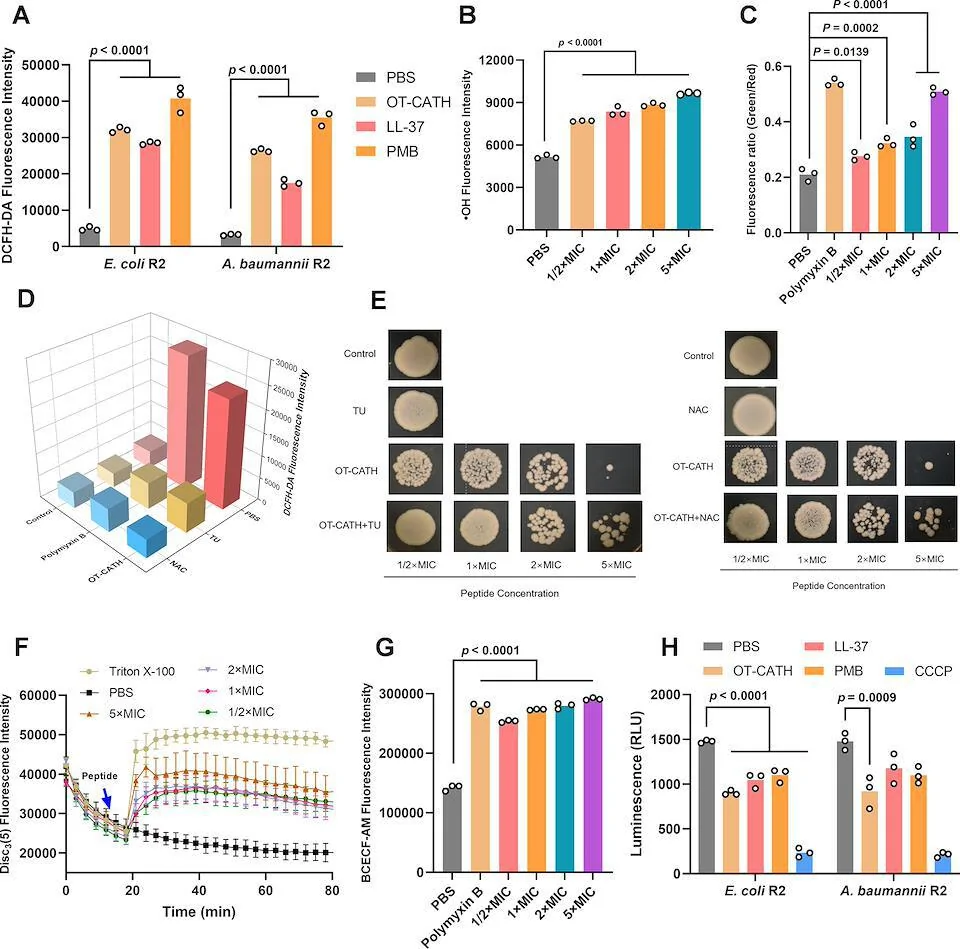
Intracellular mechanism of action of OT-CATH. (**A**) Intracellular ROS levels in ESBL-producing *E. coli* R2 and *A. baumannii* R2 after 30 min of peptide treatments, determined using the DCFH-DA probe (excitation, 488 nm; emission, 525 nm). (**B**) Intracellular .OH levels in ESBL-producing *E. coli* R2 after 30 min of peptide treatment at the indicated concentrations, determined using the HPF probe (excitation, 490 nm; emission, 515 nm). (**C**) Lipid peroxidation of ESBL-producing *E. coli* R2 after 30 min of peptide treatment at the indicated concentrations, measured using the C11-BODIPY 581/591 probe and expressed as the fluorescence ratio of oxidized (green) to reduced (red) fluorescence. (**D**) Relative DCFH-DA fluorescence intensity of ESBL-producing *E. coli* R2 following treatment in the presence or absence of TU or NAC. (**E**) Effect of TU or NAC on the antibacterial activity of OT-CATH (spotting assay). (**F**) Cytoplasmic membrane depolarization assay. The fluorescent probe 3,3′-dipropylthiadicarbocyanine iodide [DiSC3(5)] was used to detect changes in membrane potential Δψ; reduced fluorescence intensity indicates membrane depolarization. (**G**) Intracellular pH gradient (ΔpH) of ESBL-producing *E. coli* R2 after 30 min of peptide treatment, determined using the 2′,7′-bis(carboxyethyl)−5(6)-carboxyfluorescein acetate methyl ester (BCECF-AM) probe. (**H**) Intracellular ATP levels in ESBL-producing *E. coli* R2 and *A. baumannii* R2 after 30 min of peptide treatments. All experiments were performed in three biological replicates. Data are presented as mean ± SD. One-way ANOVA was used for statistical analysis, and *P*-values are indicated. Each data point represents an independent replicate.

The bacterial electron transport chain (ETC) is located in the inner membrane, and the integrity of the inner membrane is essential for the normal function of the ETC (42). Disorder or inhibition of the ETC leads to electron leakage, which reacts directly with molecular oxygen to generate excessive reactive oxygen species (ROS), thereby causing oxidative stress and bacterial death. To determine whether this response was conserved across clinically relevant gram-negative pathogens, intracellular ROS production was further evaluated in both ESBL-producing *E. coli* R2 and *A. baumannii* R2 using 2′,7′-dichlorodihydrofluorescein diacetate (DCFH-DA) (43, 44). As shown in Fig. 6A, OT-CATH markedly increased intracellular ROS levels in both ESBL-producing *E. coli* R2 and *A. baumannii* R2. Compared with the PBS group, ROS fluorescence increased by approximately sixfold in ESBL-producing *E. coli* R2 and sevenfold in *A. baumannii* R2. OT-CATH induced greater ROS accumulation than LL-37, whereas polymyxin B produced the strongest oxidative response. Further detection using the hydroxyl radical (·OH)-specific probe hydroxyphenyl fluorescein (HPF) revealed that OT-CATH induced massive intracellular ·OH generation, which peaked at 5× MIC, indicating that it can trigger a strong oxidative stress response (Fig. 6B). ROS are important intracellular signaling molecules, but excessive ROS trigger oxidative stress and attack polyunsaturated fatty acids in cell membranes, leading to lipid peroxidation, which is one of the key pathways of ROS-mediated oxidative damage (45). We therefore used C11-BODIPY581/591, a fluorescent probe that quantifies lipid peroxidation via the red/green fluorescence intensity ratio, to measure lipid peroxidation levels after OT-CATH treatment. Severe lipid peroxidation was observed following OT-CATH treatment, although the extent was lower than that of polymyxin B, which may be related to the fact that its bactericidal mechanism does not rely solely on strong oxidative stress (Fig. 6C). To confirm the functional role of ROS in OT-CATH-mediated bactericidal activity, we introduced N-acetyl-L-cysteine (NAC, a typical antioxidant) and thiourea (TU, a .OH scavenger) for intervention (Fig. 6D). The results showed that in the absence of antioxidants, almost no colonies formed in the group treated with 5× MIC OT-CATH; in contrast, the number of surviving colonies recovered significantly after the addition of NAC or TU, and this protective effect was enhanced with increasing antioxidant concentrations (Fig. 6E). Growth curve analysis (Fig. S7 and S8) further supported this finding: bacterial growth was completely inhibited in the OT-CATH monotherapy group, with OD_600_ values remaining consistently low; in the combination groups with antioxidants, however, bacteria displayed obvious “delayed growth” with a shortened lag phase and significantly restored final biomass.

The bacterial ETC drives the formation of PMF by pumping protons across the membrane. PMF consists of membrane potential (Δψ) and transmembrane proton gradient (ΔpH) (46). We used the membrane potential-sensitive dye 3,3′-dipropylthiadicarbocyanine iodide [DiSC3(5)] and the pH-sensitive probe 2′,7′-bis(carboxyethyl)−5(6)-carboxyfluorescein acetate methyl ester (BCECF-AM) to evaluate the effects of OT-CATH on Δψ and ΔpH, respectively. We found that in the untreated group, the dye accumulated in the high-potential inner membrane with fluorescence self-quenching and extremely low background fluorescence. After the addition of OT-CATH, fluorescence intensity rebounded rapidly and peaked within minutes, indicating an instantaneous collapse of membrane potential and strong depolarization that disrupted the ΔΨ component of PMF (Fig. 6F). In addition, the fluorescence intensity of BCECF-AM after OT-CATH treatment was close to that of the positive control polymyxin B even at 1× MIC, suggesting that OT-CATH can significantly disturb the bacterial proton gradient (Fig. 6G). Because disruption of the PMF is expected to impair bacterial energy metabolism (46), we further quantified intracellular ATP levels in both ESBL-producing *E. coli* R2 and *A. baumannii* R2. As shown in Fig. 6H, treatment with OT-CATH at 5× MIC significantly depleted intracellular ATP in both strains compared with the PBS control. Although ATP depletion was less pronounced than that induced by the protonophore CCCP, OT-CATH caused a substantial reduction in intracellular ATP, indicating marked impairment of bacterial energy metabolism.

In summary, OT-CATH exerts potent bactericidal activity through a cascade of events, including membrane disruption, ETC dysfunction, ROS burst, PMF collapse, and ATP depletion, while additionally imposing genotoxic stress via DNA binding, thereby establishing a multi-target and energy-dependent synergistic killing mechanism.

### Mechanism of OT-CATH with limited activity against gram-positive bacteria

**Fig 7:**
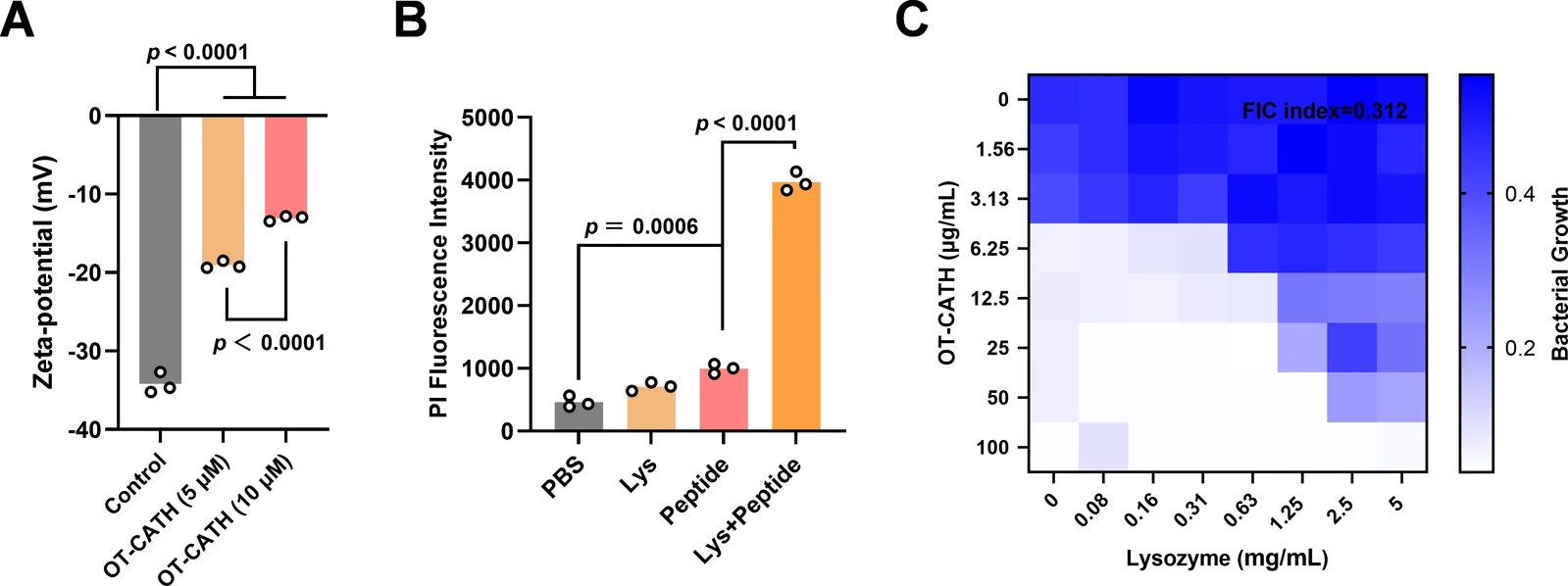
The antibacterial activity of OT-CATH against gram-positive bacteria. (**A**) Zeta potential of MRSA after treatment with different concentrations of OT-CATH. Compared with the control, treatment with 5 and 10 μM OT-CATH significantly increased the zeta potential of MRSA (*P* < 0.0001). (**B**) PI fluorescence intensity of MRSA in different treatment groups. PBS, blank control; Lys, lysozyme alone; Peptide, OT-CATH alone; and Lys + Peptide, combination of lysozyme and OT-CATH. (**C**) Checkerboard assay for the synergistic antibacterial activity of OT-CATH and lysozyme against MRSA. The color gradient represents bacterial growth (white, complete inhibition; dark blue, normal growth).

We found that the antibacterial activity of OT-CATH against gram-negative bacteria was significantly superior to that against gram-positive bacteria, which appears to contradict the broad-spectrum mechanism of most AMPs. We first detected changes in the zeta potential of methicillin-resistant *Staphylococcus aureus* (MRSA) USA300 following OT-CATH treatment to determine whether the peptide could accumulate on the bacterial surface. As shown in Fig. 7A, compared with the control group, treatment with 5 and 10 μM OT-CATH significantly increased the zeta potential of MRSA USA300 to approximately −18 and −13 mV, respectively, indicating binding of the peptide to the bacterial surface. We hypothesized that the peptide might be trapped or immobilized by the cell wall, preventing efficient contact with and disruption of the cytoplasmic membrane. Therefore, we introduced lysozyme as an agent that disrupts cell wall integrity. Figure 7B shows that there was no significant difference between the OT-CATH alone group and the control group, whereas the combination of OT-CATH with lysozyme resulted in a marked increase in PI fluorescence intensity, indicating a synergistic membrane-damaging effect. Furthermore, lysozyme pretreatment significantly increased the susceptibility of MRSA to OT-CATH (reduced MIC) and produced a strong synergistic effect (FIC index = 0.312) (Fig. 7C). These findings suggest that although OT-CATH can effectively bind to the surface of gram-positive bacteria, its antibacterial activity is limited by the structural barrier of the cell wall, which restricts access to the cytoplasmic membrane. Disruption of the cell wall restores its membrane-targeting capability, thereby explaining its apparent selectivity toward gram-negative bacteria and highlighting the critical role of envelope architecture in determining AMP efficacy.

### Pharmacokinetic properties of OT-CATH

**Fig 8:**
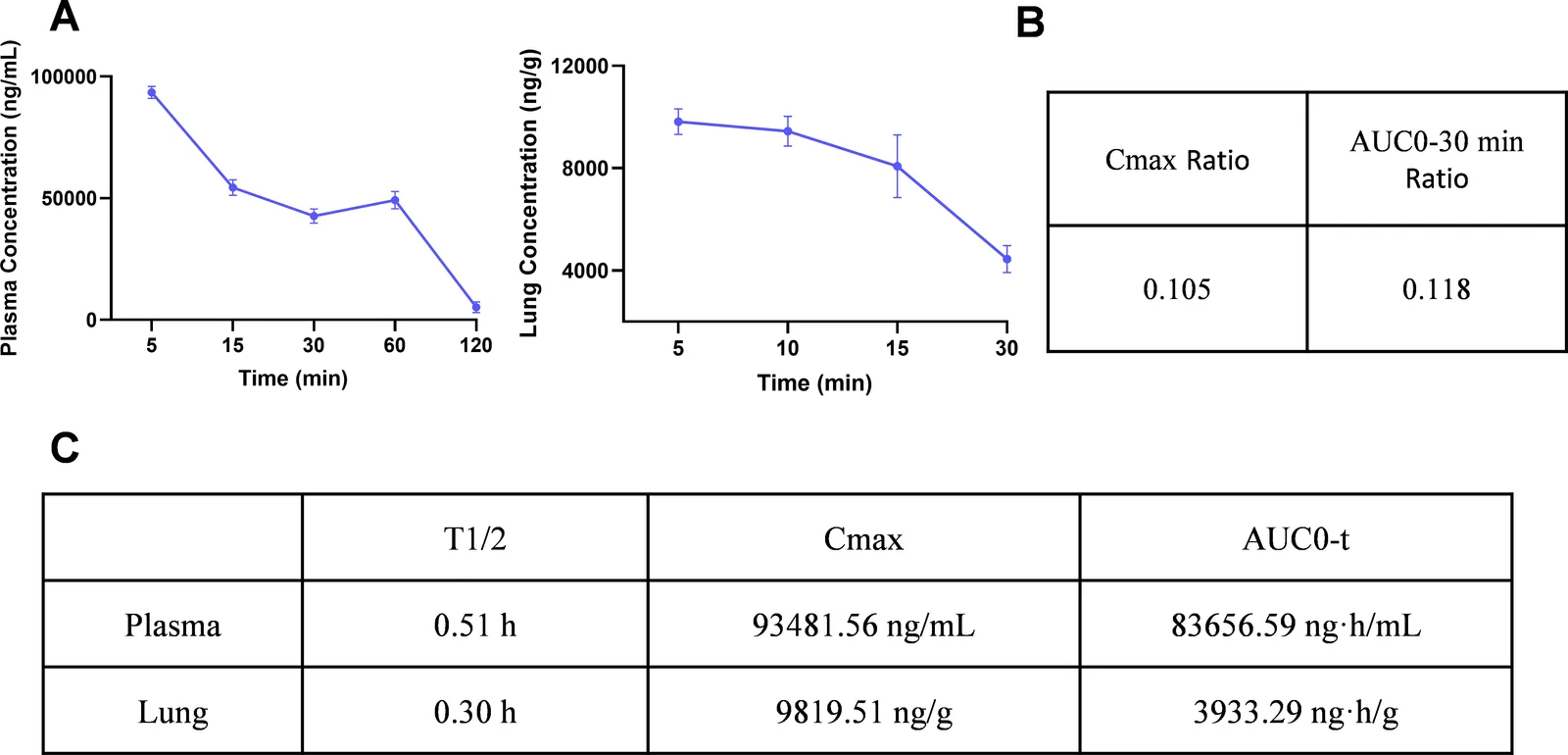
Pharmacokinetic profile and pulmonary distribution of OT-CATH following intravenous administration in mice. (**A**) Plasma and lung concentration–time profiles of OT-CATH following a single intravenous injection (10 mg/kg). OT-CATH concentrations were quantified by liquid chromatography–tandem mass spectrometry. Data are presented as mean ± SD (*n* = 3 per time point). (**B**) Lung-to-plasma distribution ratios calculated from the *C*_max_ and AUC_0–30 min_ values. (**C**) Major pharmacokinetic parameters of OT-CATH in plasma and lung tissue obtained by non-compartmental analysis.

To characterize the *in vivo* disposition of OT-CATH, we evaluated its pharmacokinetic properties following a single intravenous administration (10 mg/kg) in BALB/c mice. As shown in Fig. 8A, OT-CATH rapidly reached a peak plasma concentration (*C*_max_) of 93.48 μg/mL at 5 min, followed by a gradual decline in both plasma and lung tissue concentrations over time. OT-CATH was readily detected in the lungs throughout the sampling period, indicating rapid pulmonary distribution after systemic administration. To further assess tissue distribution, the lung-to-plasma distribution ratios based on *C*_max_ and AUC_0–30 min_ were calculated. As shown in Fig. 8B, the *C*_max_ and AUC_0–30 min_ ratios were 0.105 and 0.118, respectively, confirming that OT-CATH was efficiently distributed to lung tissue. The major pharmacokinetic parameters are summarized in Fig. 8C. OT-CATH exhibited an apparent plasma elimination half-life (*t*_1/2_) of 0.51 h, with a plasma AUC_0–*t*_ of 83,656.60 ng·h/mL. In lung tissue, the apparent *t*_1/2_ was 0.30 h, and the AUC_0–*t*_ was 3,933.29 ng·h/g.

### Therapeutic activity of OT-CATH *in vivo*

**Fig 9:**
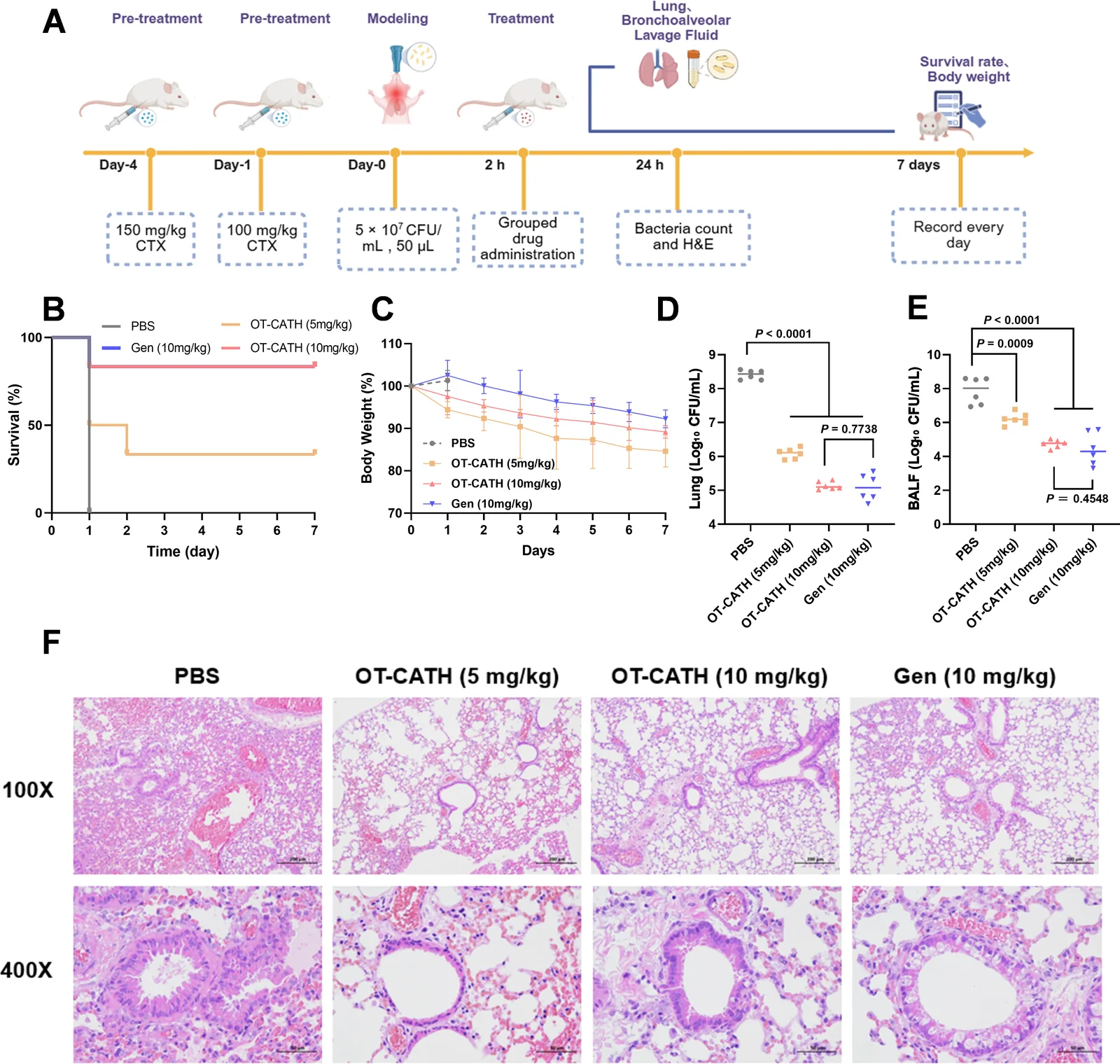
OT-CATH exhibits promising therapeutic potential in animal models. (**A**) Schematic of the mouse pneumonia infection model. CTX, cyclophosphamide; Gen, gentamicin. Mice received intraperitoneal CTX (150 mg CTX/kg of body weight on day −4 and 100 mg CTX/kg of body weight on day −1) to induce neutropenia. On day 0, pneumonia was established by slow intratracheal injection of ESBL-producing *E. coli* R2 (50 μL containing 5 × 10^7^ CFU/mL). Two hours after infection, mice were treated intraperitoneally with PBS, OT-CATH (5 or 10 mg/kg of body weight), or Gen (10 mg/kg of body weight). Bacterial burden and histopathological analyses were performed in one cohort of mice, with lung tissues and bronchoalveolar lavage fluid (BALF) collected at 24 h post-infection. Survival and body weight were monitored for 7 days in a separate cohort of mice. (**B**) Survival curves of infected mice following treatment, reflecting disease severity. (**C**) Body weight changes of infected mice within 7 days after treatment. Data are presented as percentages of initial body weight. (**D and E**) Bacterial loads in lung tissues and BALF of infected mice 24 h after OT-CATH treatment. Data are expressed as log_10_ CFU/mL. (**F**) Representative hematoxylin and eosin staining images of lung tissues 24 h after treatment. Statistical significance was determined by one-way ANOVA and two-tailed *t*-test (**D and E**). *P*-values are indicated.

Since the ESBL-producing *E. coli* R2 used in this study was isolated from sputum specimens of clinical patients, a mouse model of bacterial pneumonia was established to better simulate the actual process of clinical infection (Fig. 9A). Initially, mice were rendered immunocompromised by treatment with 150 and 100 mg cyclophosphamide/kg of body weight, followed by intratracheal instillation of 5 × 10^7^ CFU/mL ESBL-producing *E. coli* R2. Two hours after infection, mice were randomly divided into four groups: PBS group, gentamicin (10 mg/kg of body weight) group, OT-CATH (5 mg/kg of body weight) group, and OT-CATH (10 mg/kg of body weight) group. Notably, the maximum tolerated dose (MTD) of OT-CATH by intraperitoneal injection was determined to be 120 mg/kg of body weight, which is substantially higher than the treatment doses used in this study (Table S8). Following intraperitoneal injection of the same volume of agents, mortality and body weight changes were continuously monitored for 7 days as partial evaluation criteria for disease severity. Treatment with OT-CATH effectively prolonged survival and alleviated body weight loss in mice. All mice in the PBS group died within 24 h, whereas more than 80% of mice in the 10 mg/kg OT-CATH group survived until day 7 (Fig. 9B). Notably, even at a low dose of 5 mg/kg of body weight, OT-CATH effectively restricted the body weight loss of mice to within 20%, which was comparable to the efficacy of the clinically used positive control gentamicin (Fig. 9C).

To further verify the *in vivo* bactericidal activity of OT-CATH, two independent *in vivo* experiments were conducted. Both experiments adopted identical modeling and grouping protocols, with mice challenged with the same infectious dose of bacteria. After 24 h of treatment, the counts of viable bacteria in lung tissues and bronchoalveolar lavage fluid (BALF) were determined. We found that treatment with 5 mg OT-CATH/kg of body weight significantly reduced the number of viable bacteria in lung tissues, with an approximately 3-log reduction in bacterial burden (Fig. 9D). In BALF, the 5 mg/kg OT-CATH group exhibited an approximately 2-log reduction in bacterial counts, whereas the 10 mg/kg OT-CATH group showed an approximately 4-log reduction, with a therapeutic efficacy comparable to that of the positive control gentamicin (Fig. 9E), consistent with the results observed in lung tissues. These results indicate that OT-CATH exhibits superior antibacterial activity *in vivo*. Moreover, the greater efficacy of 10 mg OT-CATH/kg of body weight than that of 5 mg OT-CATH/kg of body weight in reducing bacterial counts is consistent with the *in vitro* findings.

Histological analysis further confirmed the therapeutic potential of OT-CATH. Hematoxylin and eosin staining (Fig. 9F) showed that lung tissues of mice treated with PBS exhibited typical acute inflammatory responses, including significant thickening of alveolar septa, accumulation of inflammatory exudates in some alveolar spaces, and peribronchial interstitial edema. In contrast, lung tissue structure was significantly improved in the 5 mg/kg OT-CATH treatment group. The alveolar septa thickening was reduced, the density of inflammatory cell infiltration was significantly decreased, and the alveolar spaces were basically unobstructed. Further increasing the dose to 10 mg OT-CATH/kg of body weight resulted in more significant histological changes. The alveolar structure tended to be normal, with almost no obvious aggregation of inflammatory cells. Additionally, the interstitial edema basically subsided, indicating that high-dose OT-CATH has stronger anti-inflammatory and tissue repair potential. Overall, OT-CATH exhibited effective lung tissue protective ability *in vivo*. It not only effectively eliminated pathogenic bacteria but also significantly alleviated inflammatory damage to alveoli and interstitium. This finding is consistent with its strong antibacterial activity *in vitro*, further supporting its application prospect as a potential anti-infective candidate molecule.

## DISCUSSION

With the growing crisis of antimicrobial resistance, developing drugs with novel mechanisms of action is critical to bypass established resistance pathways (11). As key effector molecules of the innate immune system, AMPs are regarded as promising alternatives to conventional antibiotics due to their multi-targeting properties (47). In this study, we identified and characterized OT-CATH from *Otariidae* species. OT-CATH exhibited potent and selective activity against multidrug-resistant gram-negative bacteria while showing favorable biosafety, stability, pharmacokinetic properties, and a low propensity for resistance development. These characteristics support its potential as a promising lead for next-generation antimicrobial therapeutics.

Different from traditional AMP discovery strategies relying on biological isolation, genome-based systematic mining provides a broader evolutionary space for identifying novel antimicrobial molecules (48). Marine mammals inhabit environments characterized by high salinity and dense gram-negative microbial communities, which may have driven the evolution of specialized innate immune peptides (49). Previous studies have identified marine mammal-derived cathelicidins with favorable salt tolerance and antimicrobial activity (50). Similarly, *Otariidae* species are continuously exposed to marine gram-negative bacteria, such as *Aeromonas hydrophila* and *Vibrio alginolyticus* (51), making them an attractive source for identifying antimicrobial peptides targeting gram-negative bacteria (52, 53). Although several marine-derived AMPs retain activity under high-salt conditions, there is currently insufficient evidence that they share conserved sequence motifs or structural signatures distinct from terrestrial AMPs. Therefore, rather than attributing the activity of OT-CATH to a marine-specific structural feature, our findings highlight marine mammalian genomes as valuable resources for antimicrobial peptide discovery.

Our mechanistic analyses suggest that OT-CATH kills bacteria through a coordinated membrane-centered cascade rather than a single molecular target. Initial interaction with outer membrane LPS facilitates membrane remodeling, leading to reduced membrane fluidity and disruption of membrane integrity (54). These events impair electron transport, collapse PMF, inhibit ATP production, and suppress energy-dependent processes such as bacterial motility and multidrug efflux (55). ETC dysfunction simultaneously promotes excessive ROS generation, resulting in lipid peroxidation and further membrane damage (56). In addition to membrane disruption, OT-CATH also binds bacterial genomic DNA, suggesting that intracellular targets may further reinforce bacterial killing. Such simultaneous disruption of multiple essential cellular processes provides a plausible explanation for the extremely low tendency of OT-CATH to induce resistance during serial passaging.

Unlike many previously reported cathelicidins, including LL-37, OT-CATH exhibited markedly greater activity against gram-negative bacteria than gram-positive bacteria (57). Our findings indicate that this selectivity is primarily determined by differences in bacterial envelope architecture rather than by a distinct membrane-targeting mechanism (54). OT-CATH readily accumulated on the MRSA surface but failed to efficiently permeabilize the cytoplasmic membrane. In contrast, partial digestion of the peptidoglycan layer with lysozyme markedly enhanced membrane permeabilization and antibacterial activity, supporting the conclusion that the thick gram-positive cell wall acts as a physical barrier limiting peptide access to the membrane (58). Nevertheless, contributions from peptide sequence composition, amphipathicity, and other physicochemical properties cannot be excluded and warrant further comparative studies.

Despite the promising antibacterial activity observed in this study, several challenges remain for the clinical development of AMPs. Similar to many peptide therapeutics, AMPs often suffer from limited systemic stability, rapid clearance, high manufacturing costs, and formulation or delivery challenges, all of which have hindered their successful clinical translation. In the present study, OT-CATH demonstrated favorable serum stability, resistance to proteolytic degradation, low hemolytic activity, limited cytotoxicity, and favorable pharmacokinetic characteristics *in vivo*, supporting its potential for further therapeutic development. Nevertheless, additional studies evaluating tissue distribution, immunogenicity, repeated-dose safety, and efficacy in multiple infection models will be required before clinical translation.

Collectively, this study identifies OT-CATH as a marine-derived cathelicidin with potent activity against multidrug-resistant gram-negative bacteria. By simultaneously disrupting bacterial membranes, collapsing energy metabolism, inducing oxidative damage, and interfering with intracellular targets, OT-CATH establishes a high evolutionary barrier to resistance development. These findings not only provide a promising antimicrobial lead but also demonstrate the value of marine mammalian genomes as an underexplored resource for next-generation anti-infective drug discovery.

## MATERIALS AND METHODS

### Identification and characterization of OT-CATH sequence

The annotated cathelicidin AMP sequences in the family *Otariidae* were systematically retrieved from the NCBI database, and representative species with close phylogenetic relationships were selected for analysis. To precisely define the mature peptide region of this family of proteins, the SignalP 6.0 online server (https://services.healthtech.dtu.dk/services/SignalP-6.0/) was used to predict signal peptide cleavage sites. Multiple sequence alignment was performed using MEGA 7.0 software to screen homologous sequences with high similarity. The distribution of four highly conserved cysteine residues in the cathelicidin domain was verified according to previously reported methods, and the mature sequence was obtained and designated as OT-CATH. The three-dimensional structure of OT-CATH was generated by SWISS-MODEL (https://swissmodel.expasy.org/). The amphiphilic character of OT-CATH was assessed using a helical wheel projection generated via the HeliQuest web server (https://heliquest.ipmc.cnrs.fr/), which illustrates the spatial segregation of hydrophobic and hydrophilic residues along its α-helical axis. Additional physicochemical parameters, including molecular weight, isoelectric point (pI), net charge at physiological pH, and overall hydrophobicity (GRAVY index), were computed using the ExPASy ProtParam tool (https://www.expasy.org/).

### Phylogenetic tree construction

Amino acid sequences of cathelicidin family AMPs from various vertebrate species, including fish, amphibians, mammals, reptiles, and birds, were retrieved from the NCBI database and strictly screened by BLASTp to ensure homology reliability. Multiple sequence alignment was performed using MEGA 7.0 software. The phylogenetic tree was constructed by the neighbor-joining method after alignment, and branch reliability was evaluated with 1,000 bootstrap replicates.

### OT-CATH synthesis and characterization

OT-CATH (RFGRLRDLIRRGRQKIAEKIQRIGQQINNIFRKLQAKKES; molecular weight, 4,863.7 Da) was chemically synthesized by GL Biochemistry Co., Ltd. (Shanghai, China) using solid-phase synthesis. The purity of the peptide (>95%) was confirmed by reversed-phase high-performance liquid chromatography (Fig. S9), and its sequence was verified by electrospray ionization mass spectrometry (Fig. S10).

### Source of bacterial strains

All standard bacterial strains and some drug-resistant bacterial strains were preserved and cultured in our laboratory under biosafety-compliant conditions, as follows: *Escherichia coli* ATCC 25922, *Klebsiella pneumoniae* ATCC 10031, *Pseudomonas aeruginosa* ATCC 27853, *Vibrio parahaemolyticus*, *Shigella dysenteriae* ATCC 12022, *Vibrio alginolyticus* 1587, *Acinetobacter baumannii* ATCC 19606, *Vibrio vulnificus*, *Staphylococcus aureus* CMCC 26003, MRSA CMCC 43300, MRSA 52, *Bacillus cereus* CMCC 63301, *Bacillus subtilis* CMCC 63501, *Clostridium perfringens* ATCC 13124, *Enterococcus faecium* 34, *Enterococcus faecalis* ATCC 29212, *Staphylococcus epidermidis* 37, MRSA ATCC 33591, MDR *S. epidermidis*, and MDR *S. aureus*. All clinical isolates were obtained from the First People’s Hospital of Zunyi.

#### Minimum inhibitory concentration assay

The *in vitro* antibacterial activity of OT-CATH and comparator antimicrobial agents was determined using a slightly modified broth microdilution method as previously described (59). Antimicrobial agents were serially twofold diluted in cation-adjusted Mueller–Hinton broth (CAMHB; Thermo Scientific Oxoid, CM0405B) to obtain the desired concentration range. The medium was supplemented with divalent cations after sterilization according to CLSI recommendations to achieve final concentrations of 20 mg/L Ca²^+^ and 10 mg/L Mg²^+^. Mid-logarithmic-phase bacterial cultures were diluted to 2 × 10^5^ CFU/mL in CAMHB, and an equal volume of bacterial suspension was added to each well. Plates were incubated at 37°C for 18 h, and the lowest antimicrobial concentration showing no visible bacterial growth was defined as the MIC. All experiments were performed in three independent biological replicates.

#### Minimum bactericidal concentration assay

The MBC of OT-CATH was determined immediately following the broth microdilution MIC assay (60). Briefly, bacterial suspensions (2 × 10^5^ CFU/mL) were incubated with serial twofold dilutions of OT-CATH in MH broth at 37°C for 18–20 h. After incubation, 50 μL aliquots were collected from wells showing no visible bacterial growth and spread onto Mueller–Hinton agar (MHA) plates. The plates were incubated at 37°C for 18–24 h, and bacterial colonies were enumerated. The MBC was defined as the lowest peptide concentration resulting in ≥99.9% killing of the initial bacterial inoculum. All experiments were performed independently in triplicate.

### Inhibition zone assay

Bacterial susceptibility to the tested antimicrobial agents was evaluated using the CLSI-standardized disk diffusion assay. Mid-logarithmic-phase bacterial cultures were adjusted to 1 × 10^8^ CFU/mL and uniformly spread onto MHA plates using sterile cotton swabs. Sterile 6-mm blank filter paper discs were placed on the agar surface, and 10 μL of OT-CATH or LL-37 solution (20 mg/mL) was applied to each disc. Commercial antimicrobial susceptibility discs (Hangzhou Microbial Reagent Co., Ltd., Hangzhou, China) were used for comparator antibiotics. The plates were incubated at 37°C for 16–18 h. The diameter of each inhibition zone, including the 6-mm disc, was measured along two perpendicular axes using a vernier caliper, and the average value was recorded. All experiments were performed in three independent biological replicates. The disk contents of the antimicrobial agents are as follows: ampicillin (10 μg), piperacillin (100 μg), cefazolin (30 μg), cefuroxime (30 μg), ceftriaxone (30 μg), ceftazidime (30 μg), cefepime (30 μg), imipenem (10 μg), meropenem (10 μg), aztreonam (30 μg), gentamicin (10 μg), tobramycin (10 μg), amikacin (30 μg), ciprofloxacin (5 μg), levofloxacin (5 μg), trimethoprim-sulfamethoxazole (1.25/23.75 μg), minocycline (30 μg), tetracycline (30 μg), colistin (10 μg), polymyxin B (300 IU), ampicillin-sulbactam (10/10 μg), piperacillin-tazobactam (100/10 μg), ticarcillin-clavulanic acid (75/10 μg), cefoperazone-sulbactam (75/75 μg), penicillin G (10 U), oxacillin (1 μg), and vancomycin (30 μg).

### Circular dichroism spectroscopy

The secondary structure of OT-CATH was analyzed by circular dichroism spectroscopy. OT-CATH was dissolved in either ultrapure water or 60 mM SDS to a final concentration of 200 μg/mL. Spectra were collected from 190 to 260 nm with a scanning speed of 100 nm/min, a bandwidth of 1 nm, and a response time of 1 s. Each spectrum was obtained as the average of three consecutive scans, and the corresponding solvent baseline was subtracted. The resulting spectra were used to evaluate the secondary structural characteristics of OT-CATH under aqueous and membrane-mimicking conditions.

#### Bactericidal kinetic assay

Mid-logarithmic phase bacteria were diluted to 1 × 10^6^ CFU/mL, mixed with equal volumes of peptide or antibiotic solutions at 5× MIC (MIC_OT-CATH_ = 18.75 µg/mL, MIC_LL-37_ = 75 µg/mL, and MIC_PMB_ = 1.17 µg/mL), and incubated at 37°C. At various time intervals, aliquots of the co-incubated suspension were collected, diluted, and plated on LB agar plates. The plates were incubated at 37°C for 18 h for bacterial colony counting (61). Time-kill curves were constructed by plotting the log_10_ CFU/mL against time. The minimum duration required to kill 99% (MDK_99_) and 99.99% (MDK_99.99_) of the initial bacterial population was determined from the time-kill curves by interpolation (62). All experiments were performed in three biological replicates.

### *In vitro* synergistic antibacterial activity of OT-CATH with antibiotics

The synergistic antibacterial effect between OT-CATH or LL-37 and antibiotics was evaluated using the checkerboard dilution method (63). Assays were carried out in sterile 96-well plates. A volume of 80 μL bacterial suspension (6 × 10^5^ CFU/mL) was added to each well. Along the horizontal direction of the plate, 10 μL of twofold serially diluted antibiotics was added, while OT-CATH was added along the vertical direction, so that each well contained equal volumes of different combinations of peptide and antibiotic concentrations with a consistent final volume. The plates were incubated statically at 37°C for 16 h. Bacterial growth was determined by visual inspection and measurement of the OD_600_ value using a microplate reader. Synergy was evaluated using the fractional inhibitory concentration index (FIC), calculated as follows: FICI_OT-CATH_ = CMIC_OT-CATH_/MIC_OT-CATH_ + CMIC_antibiotic_/MIC_antibiotic_. CMIC represents the MIC values of the agents in combination, and MIC represents the MIC values of the agents alone. A synergistic effect was defined as FIC ≤0.5.

#### Assessment of resistance development

The evolution of bacterial resistance was evaluated via a serial passaging microdilution assay (64). Exponential-phase bacterial cultures were grown in MH medium containing test agents at 1/2× MIC (MIC_OT-CATH_ = 18.75 µg/mL, MIC_LL-37_ = 75 µg/mL, MIC_PMB_ = 1.17 µg/mL, and MIC _Ciprofloxacin_ = 37.5 μg/mL) and incubated at 37°C for 12 h per passage. Cultures were serially passaged for 40 consecutive cycles; MIC measurements were performed every five passages, and the drug concentration was adjusted accordingly when elevated bacterial resistance was observed.

#### SYTO 9/PI staining assay

Mid-log-phase ESBL-producing *E. coli* R2 and *A. baumannii* R2 cultures were adjusted to 1 × 10^8^ CFU/mL and treated with OT-CATH, LL-37, or polymyxin B at the 5× MIC (MIC_OT-CATH_ = 18.75 µg/mL, MIC_LL-37_ = 75 µg/mL, and MIC_PMB_ = 1.17 µg/mL) concentrations for 1 h at 37°C. Bacterial cells were collected by centrifugation, washed twice with sterile PBS, and stained with SYTO 9 (4 μM) and propidium iodide (15 μM) for 15 min in the dark at room temperature. Fluorescence images were acquired using a confocal laser scanning microscope. Live and membrane-compromised bacteria were identified by green (SYTO 9) and red (PI) fluorescence, respectively. Fluorescence intensity was quantified using ImageJ.

#### Biofilm eradication activity assay

Biofilm eradication capacity of distinct test agents was characterized via crystal violet and 2,3,5-triphenyltetrazolium chloride staining. Logarithmic-phase bacterial suspensions were adjusted to 1 × 10^7^ CFU/mL, and 100 μL aliquots were seeded into 96-well plates, followed by 48-h incubation at 37°C to generate mature biofilms, with baseline biofilm biomass quantified at this time point. Wells were gently rinsed with PBS and incubated with individual test agents at 5× MIC (MIC_OT-CATH_ = 18.75 µg/mL, MIC_LL-37_ = 75 µg/mL, and MIC_PMB_ = 1.17 µg/mL) for an additional 24 h at 37°C. Post-treatment, biofilms were fixed with methanol, stained with 0.1% crystal violet or 0.5% TTC solution, washed with PBS to remove excess unbound stain, and air-dried at ambient temperature. Retained dye was solubilized in absolute ethanol, and absorbance was recorded at 570 and 490 nm using a microplate spectrophotometer. In parallel, recovered bacterial suspensions were serially diluted and spread onto LB agar plates to enumerate viable bacteria. All experiments were performed in three independent biological replicates.

#### Biofilm inhibition activity assay

In addition, the ability of the peptide to inhibit biofilm formation was evaluated using crystal violet and TTC staining. Logarithmic-phase bacteria were diluted to 1 × 10^7^ CFU/mL, and the wells were gently rinsed with PBS and incubated with individual test agents at 5× MIC (MIC_OT-CATH_ = 18.75 µg/mL, MIC_LL-37_ = 75 µg/mL, and MIC_PMB_ = 1.17 µg/mL) for an additional 24 h at 37°C. After incubation, the biofilms were fixed with methanol, stained with 0.1% crystal violet or 0.5% TTC solution, rinsed with PBS to remove excess dye, and air-dried at room temperature. Finally, the dye was fully dissolved in absolute ethanol, and absorbance at 570 or 490 nm was measured using a microplate reader. In parallel, recovered bacterial suspensions were serially diluted and spread onto LB agar plates to enumerate viable bacteria. All experiments were performed in three biological replicates.

#### Persister cell killing assay

Logarithmic-phase bacteria were diluted to 2 × 10^8^ CFU/mL and co-incubated with rifampicin at a concentration of 100× MIC (MIC_RIF_ = 4.69 µg/mL) at 37°C for 24 h. After incubation, the bacterial suspension was washed thoroughly with PBS to remove residual antibiotics, followed by sonication to release bacterial persisters. Peptide solutions at various concentration gradients were added, and incubation was continued at 37°C for 12 h. After incubation, an equal volume of the diluted bacterial suspension was plated on LB agar plates for colony counting (65).

#### Stability analysis

The effects of salt ions, repeated freeze–thaw cycles, and urea and protease treatments on the antibacterial activity of OT-CATH were investigated in depth. Changes in antibacterial activity were quantitatively evaluated using the MIC method (66).

For metal ion treatments, MH broth was supplemented with each metal salt at fixed final concentrations (150 mM NaCl, 4.5 mM KCl, 2 mM CaCl_2_, 1 mM MgCl_2_, 8 μM ZnCl_2_, 4 μM FeCl_3_, and 6 μM NH_4_Cl). MIC determinations were performed in ion-supplemented MH medium following a standard serial dilution setup, with unsupplemented MH medium serving as the control. Plates were incubated at 37°C with shaking for 16 h.

For Proteinase K, trypsin, urea, and serum treatments, test agents were pre-incubated with each reagent for 2 h prior to MIC measurement. For freeze–thaw assays, peptide solutions underwent repeated freeze–thaw cycles before activity testing. Antibacterial activity shifts were presented as fold increases in MIC relative to the MH control. All experiments were conducted in three independent biological replicates.

### Biosafety evaluation

To systematically assess the biosafety of OT-CATH, we evaluated its hemolytic activity (67) and cytotoxicity (68).

Fresh mouse red blood cells were collected, washed with PBS, and co-incubated with peptide solutions at various concentrations at 37°C for 30 min. After centrifugation at 1,200 × *g* at 4°C for 5 min, the absorbance of the supernatant at 540 nm was measured. Untreated red blood cells served as the negative control, and red blood cells treated with 1% Triton X-100 served as the positive control. The hemolysis rate was calculated by the following formula: Hemolysis rate (%) = (OD_540 nm_ [peptides] – OD_540 nm_ [PBS])/(OD_540 nm_ [1% Triton X-100] – OD_540 nm_ [PBS]) × 100%. All experiments were performed in three biological replicates.

HEK293, RAW264.7, BEAS-2B, and HaCaT cells were cultured in DMEM supplemented with 10% FBS and 1% penicillin–streptomycin at 37°C in a humidified incubator with 5% CO_2_. Cells in the logarithmic growth phase were seeded into 96-well plates at a density of 2 × 10^4^ cells per well and allowed to attach overnight. The culture medium was replaced with fresh DMEM, and cells were treated with serial dilutions of OT-CATH, LL-37, or polymyxin B for 24 h. Subsequently, 20 μL of MTT solution (5 mg/mL) was added to each well and incubated for 4 h. After removing the supernatant, the resulting formazan crystals were dissolved in DMSO, and absorbance was measured at 490 nm using a microplate reader. Cell viability was expressed as a percentage relative to the untreated control.

#### Membrane fluidity assay

Membrane fluidity was measured using Laurdan (Thermo Fisher Scientific, USA) (39). Logarithmic-phase bacteria were diluted to 1 × 10^8^ CFU/mL, mixed with Laurdan at a final concentration of 10 μM, and incubated at 37°C for 30 min. After incubation, drugs at various concentrations were added and treated for 30 min. PBS served as the negative control, and Triton X-100 served as the positive control. Fluorescence signals of bacteria were detected (excitation wavelength, 350 nm; emission wavelengths, 440 and 490 nm), and the generalized polarization value (GP value) was calculated according to the following formula: GP = (*I*_440_ – *I*_490_)/(*I*_440_+ *I*_490_).

#### Zeta potential assay

The change in bacterial zeta potential after peptide treatment was determined according to a previously described method (69). Logarithmic-phase bacteria were diluted to 1 × 10^8^ CFU/mL, and the membrane potential changes after treatment with different concentrations of peptide were measured using a Litesizer 500 Particle Analyzer (Anton Paar, Austria).

#### Lipid binding assay

When evaluating the antibacterial activity of OT-CATH using the checkerboard microdilution method, different concentrations of lipopolysaccharide from *Escherichia coli* O111:B4 and four acidic phospholipids, including PG, phosphatidylethanolamine, phosphatidylcholine, and cardiolipin (Sigma-Aldrich, USA), were exogenously added to determine the changes in MIC (70).

#### Isothermal titration calorimetry assay

Peptide–LPS binding affinity was determined by ITC at 25°C using a MicroCal instrument (Malvern, USA). Measurements were performed in ITC buffer containing 20 mM Tris–HCl (pH 7.5) and 150 mM NaCl. LPS from *E. coli* O55:B5 was titrated into the calorimetric cell containing OT-CATH at a molar ratio of 10:1. A total of 20 sequential injections were performed with an interval of 200 s between each injection to ensure baseline equilibration. Raw data were integrated and fitted using Origin software with a single-site binding model to obtain the equilibrium dissociation constant (*K_d_*).

#### Outer membrane permeability assay

The effect of the peptide on bacterial outer membrane permeability was investigated using the fluorescent dye 1-N-phenylnaphthylamine (Sigma-Aldrich, USA) (71). Logarithmic-phase bacteria were diluted to 1 × 10^8^ CFU/mL, mixed with test agents at 5× MIC (MIC_OT-CATH_ = 18.75 µg/mL, MIC_LL-37_ = 75 µg/mL, and MIC_PMB_ = 1.17 µg/mL) and NPN dye (final concentration, 10 μM) in a 96-well black plate, and co-incubated at 37°C for 30 min. Fluorescence intensity was measured immediately after incubation (excitation wavelength, 350 nm; emission wavelength, 420 nm). PBS served as the negative control, and polymyxin B served as the positive control.

#### Inner membrane permeability assay

Inner membrane permeability was assessed using propidium iodide (Sigma-Aldrich, USA) (72). Logarithmic-phase bacteria were diluted to 1 × 10^8^ CFU/mL, mixed with test agents at 5× MIC (MIC_OT-CATH_ = 18.75 µg/mL, MIC_LL-37_ = 75 µg/mL, and MIC_PMB_ = 1.17 µg/mL) and PI dye (final concentration, 5 μM) in a 96-well black plate, and co-incubated at 37°C for 30 min. Fluorescence intensity was measured immediately after incubation (excitation wavelength, 535 nm; emission wavelength, 615 nm). PBS served as the negative control, and Triton X-100 served as the positive control.

#### Bacterial intracellular content leakage assay

The assay was performed with minor modifications according to a previously described method (65). Logarithmic-phase bacteria were diluted to 1 × 10^9^ CFU/mL and incubated with different concentrations of peptide solution at 37°C. At designated time intervals, aliquots of the same volume were collected, and the concentrations of DNA, RNA, and protein in the samples were measured using a NanoDrop One microvolume spectrophotometer (Thermo Scientific, USA).

#### ONPG assay

Logarithmic-phase bacteria were diluted to 1 × 10^8^ CFU/mL and mixed with different concentrations of peptide solution and O-nitrophenyl-β-D-galactopyranoside (Aladdin, China) at a final concentration of 1 mg/mL, followed by incubation at 37°C. Then, 100 μL of the mixture was added to each well, and the absorbance at 420 nm was measured every 5 min for a total of 120 min (73).

#### SEM characterization

Logarithmic-phase bacteria were diluted to 5 × 10^8^ CFU/mL and incubated with peptide solution at 37°C for 1 h. The mixture was then fixed overnight with 2.5% glutaraldehyde at 4°C, washed with PBS, and treated sequentially with a graded ethanol series (30%, 50%, 70%, 90%, and 100%) for 10 min each, followed by treatment with absolute ethanol:tert-butanol = 1:1 (vol/vol) and pure tert-butanol for 10 min each. The final bacterial suspension in tert-butanol was dropped onto clean glass slides, air-dried, and sputter-coated with gold. Images were captured using a Hitachi S-4800 scanning electron microscope (Hitachi, Japan).

#### DNA gel retardation assay

DNA gel retardation assay was performed to detect the binding affinity of the peptide to bacterial genomic DNA (74). Bacterial genomic DNA was extracted using the TIANamp Bacteria DNA Kit (TIANGEN, China), mixed with different concentrations of peptide solution, and incubated at 37°C for 30 min. An appropriate amount of each sample was subjected to 1% agarose gel electrophoresis. DNA mobility was visualized using a Bio-Rad imaging system (Bio-Rad, USA).

#### Detection of reactive oxygen species

Intracellular ROS levels in bacteria were detected using the 2′,7′-dichlorodihydrofluorescein diacetate (Beyotime, China) probe (75). Logarithmic-phase bacteria were diluted to 1 × 10^8^ CFU/mL and co-incubated with 10 μM DCFH-DA at 37°C for 30 min. After incubation, excess probe was removed by washing, and bacteria were treated with test agents at 5× MIC (MIC_OT-CATH_ = 18.75 µg/mL, MIC_LL-37_ = 75 µg/mL, and MIC_PMB_ = 1.17 µg/mL) for 30 min. Fluorescence intensity was then measured (excitation wavelength, 488 nm; emission wavelength, 525 nm).

#### Measurement of hydroxyl radicals(·OH)

Bacterial .OH levels were evaluated using the hydroxyphenyl fluorescein (Macklin, China) probe with minor modifications to a previously described method (76). Logarithmic-phase bacteria were diluted to 1 × 10^8^ CFU/mL and co-incubated with the HPF probe for 30 min, followed by the addition of various concentrations of peptide and further incubation for 1 h. Fluorescence intensity was measured immediately after incubation (excitation wavelength, 490 nm; emission wavelength, 515 nm).

#### Lipid peroxidation assay

Bacterial lipid peroxidation levels were detected using the C11-BODIPY 581/591 probe (Solarbio, China) (77). Logarithmic-phase bacteria were diluted to 5 × 10^8^ CFU/mL and incubated with 10 μM C11-BODIPY 581/591 at room temperature for 30 min to allow probe membrane incorporation. After incubation, unbound probe was removed by washing, and different concentrations of drugs were added, followed by another 30-min incubation. Fluorescence intensities of two groups were measured separately after incubation: I₁ at 488 nm excitation/510 nm emission; I₂ at 581 nm excitation/591 nm emission. Lipid peroxidation levels were quantified by calculating the ratio of I₁/I₂ fluorescence intensity.

#### ROS inhibitor assay

N-acetylcysteine and thiourea were used to investigate the significance of ROS inhibitors in the function of the OT-CATH peptide. Briefly, NAC and TU were added to mixtures of peptide and bacterial cultures. The effects of inhibitors on the growth of peptide-exposed bacteria were analyzed by colony counting and bacterial growth curve determination.

#### ATP level assay

Intracellular ATP levels of bacteria exposed to the peptide were detected using an ATP Assay Kit (Beyotime, China) (78). Briefly, logarithmic-phase bacteria were diluted to 5 × 10^8^ CFU/mL and co-incubated with test agents at 5× MIC (MIC_OT-CATH_ = 18.75 µg/mL, MIC_LL-37_ = 75 µg/mL, and MIC_PMB_ = 1.17 µg/mL) for 30 min. After centrifugation at 12,000 × *g* at 4°C for 5 min, the supernatant was collected for extracellular ATP measurement, and the bacterial pellet was lysed with lysozyme for intracellular ATP determination.

#### Cytoplasmic membrane depolarization assay

The extent of bacterial membrane depolarization was evaluated using the membrane potential-sensitive fluorescent dye 3,3′-dipropylthiadicarbocyanine iodide (Sigma-Aldrich, USA) (79). Logarithmic-phase bacteria were diluted to 3 × 10^7^ CFU/mL and incubated with DiSC3(5) at 37°C. Fluorescence intensity was recorded (excitation wavelength, 622 nm; emission wavelength, 670 nm) until stabilization. Different drugs were then added, and detection was continued for 60 min.

### Intracellular pH gradient measurement

The pH-sensitive fluorescent probe 2′,7′-bis(carboxyethyl)−5(6)-carboxyfluorescein acetate methyl ester (Beyotime, China) was used (80). Logarithmic-phase bacteria were diluted to 1 × 10^8^ CFU/mL and co-incubated with different concentrations of peptide solution and 5 μM BCECF-AM at 37°C for 30 min. Fluorescence intensity was measured immediately after incubation (excitation wavelength, 488 nm; emission wavelength, 535 nm).

### Efflux pump activity assay

Bacterial efflux pump activity was evaluated using tetracycline and Rhodamine 6G with minor modifications to previously described methods (30, 31). Logarithmic-phase bacteria were diluted to 5 × 10^8^ CFU/mL and co-incubated with tetracycline or Rhodamine 6G at 37°C for 30 min. After incubation, unabsorbed substrates were removed by washing, different concentrations of drugs were added, and 2% glucose was supplemented to initiate efflux. Fluorescence intensity was measured immediately. For tetracycline, the excitation wavelength was 405 nm, and the emission wavelength was 535 nm; for Rhodamine 6G, the excitation wavelength was 525 nm, and the emission wavelength was 560 nm.

### Swimming motility assay

The swimming motility assay was performed with minor modifications as previously described (81, 82). Briefly, swimming plates were prepared by supplementing LB medium with 0.3% (wt/vol) agar. After cooling the medium to approximately 50°C, OT-CATH, LL-37, or polymyxin B was added at the indicated sub-MIC concentrations and mixed thoroughly before pouring onto the plates. Logarithmic-phase bacterial cultures were adjusted to 1 × 10^8^ CFU/mL, and 2 μL of the bacterial suspension was inoculated onto the center of each plate. The plates were incubated at 37°C for 16 h, and swimming motility was quantified by measuring the colony diameter.

### Pharmacokinetic analysis

BALB/c mice were intravenously administered OT-CATH at a single dose of 10 mg/kg of body weight via the tail vein. For plasma pharmacokinetic analysis, blood samples were collected at 5, 15, 30, 60, and 120 min after dosing (*n* = 3 mice per time point). Plasma was separated by centrifugation and stored at −80°C until analysis.

For lung tissue distribution analysis, mice were euthanized at 5, 10, 15, and 30 min after dosing (*n* = 3 mice per time point). Lungs were excised, rinsed briefly with cold PBS to remove residual blood, weighed, snap-frozen in liquid nitrogen, and stored at −80°C until analysis.

OT-CATH concentrations in plasma and lung tissue were determined by liquid chromatography–tandem mass spectrometry. Pharmacokinetic parameters, including the elimination half-life (*t*_1/2_), maximum concentration (*C*_max_), and area under the concentration–time curve (AUC), were calculated by non-compartmental analysis. Pulmonary distribution was evaluated using the lung-to-plasma ratios of *C*_max_ and AUC_0–30 min_.

### Animals and acute toxicity assessment

The MTD of OT-CATH by intraperitoneal injection was determined in a dose-escalation acute toxicity study. BALB/c mice (*n* = 6 per group) were administered a single intraperitoneal injection of OT-CATH at doses ranging from 80 to 120 mg/kg of body weight. Animals were closely observed for 7 days, and any clinical signs of toxicity (e.g., piloerection, reduced locomotion, hunched posture, body weight loss greater than 20%, or respiratory distress) and mortality were recorded daily. The MTD was defined as the highest dose at which no death or severe toxicity occurred.

### *In vivo* therapeutic efficacy evaluation of OT-CATH

To evaluate the *in vivo* antibacterial efficacy of OT-CATH, specific pathogen-free BALB/c mice aged 6–8 weeks, weighing 20 ± 2 g, with equal gender distribution were used. Animals were acclimated for at least 24 h prior to experiments.

Mice were injected intraperitoneally with cyclophosphamide dissolved in sterile PBS at doses of 150 and 100 mg/kg of body weight on days 4 and 1 before bacterial infection, respectively, to induce neutropenia. ESBL-producing *E. coli* R2 was cultured to the exponential growth phase, harvested by centrifugation at 7,000 rpm for 5 min, washed twice with sterile PBS, and resuspended in sterile PBS. The bacterial suspension was adjusted to the desired concentration according to the optical density at 600 nm. On the day of infection, mice were anesthetized with 1.25% tribromoethanol and intubated intratracheally, followed by slow intratracheal injection of 50 μL bacterial suspension containing 5 × 10^7^ CFU to establish the pneumonia model. Thirty minutes after infection, mice were randomly assigned to four groups: Group 1, PBS (*n* = 6); Group 2, gentamicin (10 mg/kg of body weight) (*n* = 6); Group 3, OT-CATH (5 mg/kg of body weight) (*n* = 6); and Group 4, OT-CATH (10 mg/kg of body weight) (*n* = 6). All treatments were administered intraperitoneally at equal volumes. Body weight and survival were monitored daily for seven consecutive days. As all mice in the PBS group succumbed to infection within 24 h, no body weight measurements were available beyond this time point.

A second independent cohort of mice was infected and treated using the same protocol for bacterial burden analysis (*n* = 6 per group). Mice that survived to 24 h post-infection were euthanized for tissue collection, whereas animals that died before the scheduled endpoint were processed immediately after death. Lung tissues were harvested and homogenized, and bronchoalveolar lavage fluid was collected. Lung homogenates and BALF were serially diluted, plated on LB agar, and incubated for bacterial enumeration. Bacterial burden was determined by counting CFUs using an automatic colony counter (DTS3, Ze Xi Biotechnology, Hangzhou) (83).

### Statistical analysis

All experiments were performed with at least three independent biological replicates. Data are presented as mean ± standard deviation. Statistical significance was determined by one-way analysis of variance, followed by Tukey’s *post hoc* test for multiple comparisons, or by two-tailed unpaired Student’s *t*-test for comparisons between two groups. Analyses were conducted using GraphPad Prism 9.0 and Origin 2021.

## SUPPLEMENTAL MATERIAL

Supplemental material aac.01026-26-s0001.docx Tables S1 to S8; Fig. S1 to S10.

ASM does not own the copyrights to Supplemental Material that may be linked to, or accessed through, an article. The authors have granted ASM a non-exclusive, world-wide license to publish the Supplemental Material files. Please contact the corresponding author directly for reuse.
