# Supplementary material for: OT-CATH, a conserved antimicrobial peptide in Otariidae species, exhibits potent activity against gram-negative bacteria
Source: Antimicrob Agents Chemother. 2026 Oct 1;70(11):e01026-26. doi: 10.1128/aac.01026-26 (PMC13731187; doi:10.1128/aac.01026-26)
Supplement: Supplementary file 1 [file aac.01026-26-s0001.docx]

**Supplementary Information**

**OT-CATH, a conserved antimicrobial peptide in *Otariidae* species, exhibits potent activity against Gram-negative bacteria**

Yixiao Song^1,2^, Yufeng Ren^1,2^, Juan Wang^1,3^, Zifan Ye^1,4^, Chenchen Shi^1,2^, Xiangjin Kong^1,2^, Xudong Jiao^1,2^, Jingjing Zhang^1,2^, Yipeng Wang^1,2,^*

^1^Yantai Institute of Coastal Zone Research, Chinese Academy of Sciences, Yantai, Shandong 264003, China.

^2^University of Chinese Academy of Sciences, Beijing 100049, China.

^3^Research Institute of Marine Traditional Chinese Medicine (Qingdao Academy of Chinese Medical Sciences), Shandong University of Traditional Chinese Medicine, Qingdao 266112, China.

^4^Department of Biopharmaceutical Sciences, College of Pharmaceutical Sciences, Soochow University, Suzhou, Jiangsu 215123, China.

*Corresponding author:

Yipeng Wang, Yantai Institute of Coastal Zone Research, Chinese Academy of Sciences, Yantai, Shandong 264003, China. Email: [ypwang@yic.ac.cn](mailto:ypwang@yic.ac.cn).

**Table S1. Clinical bacterial strains used in this study: sources, resistance phenotypes, and resistance gene profiles.**

| **Species** | **Source** | **Resistance type** | **Resistance profile (by class)** | **Resistance genes** |
| --- | --- | --- | --- | --- |
| *E. coli* R1 | Sputum | ESBL-producing | Penicillins, Cephalosporins | *blaCTX-M* |
| *E. coli* R2 | Sputum | ESBL-producing | Penicillins, Cephalosporins, Fluoroquinolones | *blaCTX-M* |
| *E. coli* R3 | Urine | ESBL-producing | Penicillins, Cephalosporins, Monobactams, Fluoroquinolones, Sulfonamides | *blaCTX-M* |
| *A. baumannii* R1 | Sputum | XDR | Penicillins, Cephalosporins, Carbapenems, Aminoglycosides, Fluoroquinolones, Sulfonamides | *blaTEM-1, blaOXA-23* |
| *A. baumannii* R2 | Sputum | XDR | Penicillins, Cephalosporins, Carbapenems, Aminoglycosides, Fluoroquinolones, Sulfonamides | *blaTEM-1, blaOXA-23* |
| *A. baumannii* R3 | Sputum | XDR | Penicillins, Cephalosporins, Carbapenems, Aminoglycosides, Fluoroquinolones, Sulfonamides, Tetracyclines | *blaTEM-1, blaOXA-23* |
| *P. aeruginosa* R1 | Sputum | MDR | Penicillins, Cephalosporins, Monobactams, Carbapenems, Fluoroquinolones | *blaOXA-10-like, blaVIM-2* |
| *P. aeruginosa* R2 | Sputum | MDR | Penicillins, Cephalosporins, Monobactams | *blaOXA-10-like* |
| *P. aeruginosa* R3 | Sputum | XDR | Penicillins, Cephalosporins, Monobactams, Carbapenems, Aminoglycosides | *blaOXA-10-like, blaVIM-2* |
| *K. pneumoniae* R1 | Sputum | CRE, MDR | Cephalosporins, Monobactams, Carbapenems, Aminoglycosides, Fluoroquinolones, Sulfonamides, Cephamycins | *blaKPC-2, blaCTX-M* |
| *K. pneumoniae* R3 | Sputum | CRE, MDR | Cephalosporins, Monobactams, Carbapenems, Aminoglycosides, Fluoroquinolones, Sulfonamides, Cephamycins | *blaKPC-2, blaCTX-M* |
| *S. aureus* R1 | Sputum | MRSA | Penicillins, Macrolides, Lincosamides | *mecA, erm(C)* |
| *S. aureus* R2 | Sputum | MRSA | Penicillins, Anti-staphylococcal penicillins, Macrolides, Lincosamides | *mecA, erm(C)* |
| *S. aureus* R3 | Pus | MRSA | Penicillins, Anti-staphylococcal penicillins, Macrolides, Lincosamides | *mecA, erm(C)* |
| *S. epidermidis* R1 | Blood | MRSE, MDR | Penicillins, Anti-staphylococcal penicillins, Fluoroquinolones | *mecA, gyrA* |
| *S. epidermidis* R2 | Blood | MRSE, MDR | Penicillins, Anti-staphylococcal penicillins, Aminoglycosides, Fluoroquinolones, Macrolides, | *mecA, gyrA* |
| *S. epidermidis* R3 | Pus | MSSE | Penicillins, Macrolides | *erm(C)* |

**Table S2. Antimicrobial susceptibility of Gram‑negative bacteria used in this study.**

|  | ***E. coli* ATCC 25922** | ***E. coli* R1** | ***E. coli* R2** | ***E. coli* R3** | ***A. baumannii* ATCC 19606** | ***A. baumannii* R1** | ***A. baumannii* R2** | ***A. baumannii* R3** | ***P. aeruginosa* ATCC 27853** | ***P. aeruginosa* R1** | ***P. aeruginosa* R2** | ***P. aeruginosa* R3** | ***K. pneumoniae* ATCC 10031** | ***K. pneumoniae* R1** | ***K. pneumoniae* R2** |
| --- | --- | --- | --- | --- | --- | --- | --- | --- | --- | --- | --- | --- | --- | --- | --- |
| Ampicillin | S | R | R | R |  |  |  |  |  |  |  |  |  |  |  |
| Piperacillin |  |  |  |  | S | R | R | R | S | R | R | R |  |  |  |
| Cefazolin | S | R | R | R |  |  |  |  |  |  |  |  | S | R | R |
| Cefotetan | S | S | S | S |  |  |  |  |  |  |  |  | S | R | R |
| Cefuroxime | S | R | R | R |  |  |  |  |  |  |  |  | S | R | R |
| Ceftriaxone | S | R | R | R |  |  |  |  |  |  |  |  | S | R | R |
| Ceftazidime | S | S | R | R | S | R | R | R | S | R | R | R | S | R | R |
| Cefepime | S | R | R | R | S | R | R | R | S | S | S | S | S | R | R |
| Imipenem | S | S | S | S | S | R | R | R | S | R | S | R | S | R | R |
| Meropenem | S | S | S | S | S | R | R | R | S | R | S | R | S | R | R |
| Aztreonam | S | S | R | R |  |  |  |  | S | R | R | R | S | R | R |
| Gentamicin | S | S | S | S | S | R | R | R | S | S | S | S | S | R | R |
| Tobramycin | S | S | S | S | S | R | R | R | S | S | S | S | S | R | R |
| Amikacin | S | S | S | S | S | R | R | R | S | S | S | R | S | R | R |
| Ciprofloxacin | S | S | R | R | S | R | R | R | S | R | S | S | S | R | R |
| Levofloxacin | S | I | R | R | S | I | I | R | S | R | S | S | S | R | R |
| Trimethoprim-Sulfamethoxazole | S | S | S | R |  | R | R | R |  |  |  |  | S | R | R |
| Minocycline |  |  |  |  | S | I | I | R |  |  |  |  |  |  |  |
| Tigecycline |  |  |  |  | S | S | S | S |  |  |  |  |  |  |  |
| Ampicillin-Sulbactam | S | I | R | S | S | R | R | R |  |  |  |  | S | R | R |
| Piperacillin-Tazobactam | S | S | S | S | S | R | R | R | S | R | R | R | S | R | R |
| Ticarcillin-Clavulanic Acid |  |  |  |  |  |  |  |  | S | R | R | R |  |  |  |
| Cefoperazone-Sulbactam | S | S | S | S | S | R | I | R | S | R | R | R | S | R | R |

S = susceptible; I = intermediate; R = resistant; ND = not determined.

**Table S3. MICs (µg/mL) of OT‑CATH against Gram‑negative bacteria.**

|  | ***E. coli* ATCC 25922** | ***E. coli* R1** | ***E. coli* R2** | ***E. coli* R3** | ***A. baumannii* ATCC 19606** | ***A. baumannii* R1** | ***A. baumannii* R2** | ***A. baumannii* R3** | ***P. aeruginosa* ATCC 27853** | ***P. aeruginosa* R1** | ***P. aeruginosa* R2** | ***P. aeruginosa* R3** | ***K. pneumoniae* ATCC 10031** | ***K. pneumoniae* R1** | ***K. pneumoniae* R2** |
| --- | --- | --- | --- | --- | --- | --- | --- | --- | --- | --- | --- | --- | --- | --- | --- |
| OT-CATH | 9.38 | 18.75 | 18.75 | 37.5 | 18.75 | 75 | .37.5 | 37.5 | 75 | 75 | 75 | 75 | 18.75 | 37.5 | 37.5 |
| LL37 | 75 | 75 | 75 | 75 | 75 | 75 | 75 | 75 | >100 | >100 | >100 | >100 | >100 | >100 | >100 |
| Ampicillin | 2.34 | >100 | >100 | >100 |  |  |  |  |  |  |  |  |  |  |  |
| Piperacillin |  |  |  |  | 9.38 | >100 | ·· | >100 | 2.34 | >100 | >100 | >100 |  |  |  |
| Cefazolin | 1.17 | >100 | >100 | >100 |  |  |  |  |  |  |  |  | 2.34 | 75 | 75 |
| Cefuroxime | 4.69 | >100 | >100 | >100 |  |  |  |  |  |  |  |  | 2.34 | >100 | >100 |
| Ceftriaxone | 0.59 | >100 | >100 | >100 |  |  |  |  |  |  |  |  | 0.59 | >100 | >100 |
| Ceftazidime | 0.59 | 0.59 | >100 | >100 | 4.69 | >100 | >100 | >100 | 1.17 | >100 | >100 | 37.5 | 0.59 | >100 | >100 |
| Cefepime | 0.59 | >100 | >100 | >100 | 4.69 | 37.5 | 75 | 75 | 2.34 | 4.69 | 4.69 | 4.69 | 1.17 | >100 | >100 |
| Imipenem | 0.29 | 0.59 | 0.59 | 0.59 | 0.15 | 37.5 | 75 | 37.5 | 1.17 | 75 | 0.59 | 75 | 0.59 | >100 | 75 |
| Meropenem | 0.59 | 0.29 | 0.29 | 0.59 | 0.59 | 75 | 75 | 75 | 0.15 | 75 | 0.15 | 75 | 0.29 | >100 | 75 |
| Aztreonam | 0.59 | 1.17 | 18.75 | 18.75 |  |  |  |  | 1.17 | 75 | 37.5 | 75 | 0.29 | >100 | >100 |
| Gentamicin | 1.17 | 1.17 | 1.17 | 0.59 | 1.17 | 18.75 | 37.5 | 18.75 | 0.59 | 1.17 | 0.59 | 0.59 | 1.17 | 75 | 75 |
| Tobramycin | 1.17 | 1.17 | 0.59 | 0.59 | 1.17 | 75 | 18.75 | 75 | 0.59 | 0.59 | 0.59 | 0.59 | 1.17 | 75 | 75 |
| Amikacin | 2.34 | 1.17 | 1.17 | 1.17 | 1.17 | >100 | >100 | >100 | 4.69 | 4.69 | 1.17 | >100 | 2.34 | >100 | >100 |
| Ciprofloxacin | 0.07 | 0.15 | 37.5 | 75 | 0.59 | 37.5 | 75 | >100 | 0.15 | 75 | 0.15 | 0.15 | 0.07 | 37.5 | 37.5 |
| Levofloxacin | 0.07 | 1.17 | 75 | 75 | 1.17 | 4.69 | 4.69 | 75 | 0.290 | 75 | 0.290 | 0.15 | 0.29 | 75 | 75 |
| Trimethoprim-Sulfamethoxazole | ≤1/19 | ≤1/19 | ≤1/19 | ≥16/304 | ≤1/19 | ≥16/304 | ≥16/304 | ≥16/304 |  |  |  |  | ≤1/19 | ≥16/304 | ≥16/304 |
| Minocycline |  |  |  |  | 0.29 | 2.34 | 2.34 | 9.38 |  |  |  |  |  |  |  |
| Tigecycline |  |  |  |  | 0.59 | 0.59 | 1.17 | 1.17 |  |  |  |  |  |  |  |
| Colistin | 1.17 | 2.34 | 2.34 | 1.17 | 1.17 | 0.59 | 2.34 | 2.34 | 1.17 | 2.34 | 2.34 | 2.34 | 1.17 | 2.34 | 1.17 |
| Polymyxin B | 1.17 | 1.17 | 1.17 | 1.17 | 1.17 | 1.17 | 2.34 | 1.17 | 1.17 | 1.17 | 1.17 | 1.17 | 1.17 | 1.17 | 1.17 |
| Ampicillin-Sulbactam | <=8/4 | 16/8 | >=32/16 | <=8/4 | <=8/4 | >=32/16 | >=32/16 | >=32/16 |  |  |  |  | <=8/4 | >=32/16 | >=32/16 |
| Piperacillin-Tazobactam | <=8/4 | <=8/4 | <=8/4 | <=8/4 | <=8/4 | >=64/4 | >=64/4 | >=64/4 | <=8/4 | >=128/4 | >=128/4 | >=128/4 | <=8/4 | >=128/4 | >=128/4 |
| Ticarcillin-Clavulanic Acid |  |  |  |  |  |  |  |  | <=16/2 | >=128/2 | >=128/2 | >=128/2 |  |  |  |
| Cefoperazone-Sulbactam | <=16 | <=16 | <=16 | <=16 | <=16 | >=64 | 32 | >=64 | <=16 | >=64 | >=64 | >=64 | <=16 | >=64 | >=64 |

**Table S4. Zone of inhibition (mm) of OT‑CATH against Gram‑negative bacteria determined.**

|  | ***E. coli* ATCC 25922** | ***E. coli* R1** | ***E. coli* R2** | ***E. coli* R3** | ***A. baumannii* ATCC 19606** | ***A. baumannii* R1** | ***A. baumannii* R2** | ***A. baumannii* R3** | ***P. aeruginosa* ATCC 27853** | ***P. aeruginosa* R1** | ***P. aeruginosa* R2** | ***P. aeruginosa* R3** | ***K. pneumoniae* ATCC 10031** | ***K. pneumoniae* R1** | ***K. pneumoniae* R2** |
| --- | --- | --- | --- | --- | --- | --- | --- | --- | --- | --- | --- | --- | --- | --- | --- |
| OTCATH | 14 | 14 | 14 | 13 | 13 | 12 | 12 | 7 | 12 | 12 | 12 | 6 | 13 | 12 | 12 |
| LL37 | 13 | 12 | 12 | 9 | 6 | 12 | 12 | 6 | 7 | 7 | 6 | 6 | 6 | 7 | 7 |
| Ampicillin | 18 | 6 | 6 | 6 |  |  |  |  |  |  |  |  |  |  |  |
| Piperacillin |  |  |  |  | 23 | 6 | 6 | 6 | 23 | 7 | 6 | 6 |  |  |  |
| Cefazolin | 26 | 6 | 6 | 6 |  |  |  |  |  |  |  |  | 25 | 6 | 6 |
| Cefuroxime | 24 | 6 | 6 | 6 |  |  |  |  |  |  |  |  | 30 | 6 | 6 |
| Ceftriaxone | 29 | 7 | 6 | 6 |  |  |  |  |  |  |  |  | 30 | 8 | 6 |
| Ceftazidime | 25 | 23 | 7 | 7 | 20 | 6 | 6 | 6 | 21 | 6 | 6 | 6 | 24 | 6 | 6 |
| Cefepime | 30 | 7 | 7 | 6 | 20 | 10 | 6 | 6 | 28 | 21 | 22 | 21 | 30 | 6 | 7 |
| Imipenem | 26 | 26 | 24 | 23 | 32 | 6 | 6 | 6 | 27 | 6 | 25 | 6 | 25 | 8 | 6 |
| Meropenem | 27 | 26 | 27 | 37 | 25 | 7 | 7 | 6 | 28 | 6 | 27 | 6 | 25 | 6 | 8 |
| Aztreonam | 25 | 23 | 10 | 10 |  |  |  |  | 26 | 10 | 9 | 9 | 26 | 6 | 7 |
| Gentamicin | 20 | 20 | 21 | 21 | 20 | 10 |  | 10 | 19 | 18 | 18 | 18 | 19 | 6 | 6 |
| Tobramycin | 20 | 20 | 21 | 19 | 18 | 10 | 9 | 9 | 23 | 24 | 24 | 24 | 20 | 6 | 6 |
| Amikacin | 20 | 19 | 18 | 18 | 18 | 8 | 7 | 6 | 23 | 24 | 21 | 7 | 19 | 6 | 7 |
| Ciprofloxacin | 32 | 31 | 6 | 6 | 25 | 6 | 6 | 6 | 30 | 6 | 30 | 28 | 35 | 6 | 6 |
| Levofloxacin | 29 | 17 | 6 | 6 | 26 | 16 | 16 | 6 | 28 | 7 | 28 | 29 | 28 | 7 | 7 |
| Trimethoprim-Sulfamethoxazole | 25 | 26 | 27 | 10 | 25 | 6 | 6 | 6 |  |  |  |  | 27 | 6 | 6 |
| Minocycline |  |  |  |  | 25 | 15 | 15 | 7 |  |  |  |  |  |  |  |
| Tetracycline |  |  |  |  | 27 | 25 | 26 | 26 |  |  |  |  |  |  |  |
| Colistin | 13 | 13 | 14 | 13 | 14 | 14 | 13 | 13 | 13 | 14 | 12 | 13 | 15 | 15 | 15 |
| Polymyxin B | 14 | 14 | 15 | 15 | 15 | 16 | 14 | 14 | 15 | 16 | 15 | 16 | 14 | 14 | 13 |
| Ampicillin-Sulbactam | 26 | 18 | 6 | 27 | 26 | 8 | 10 | 8 |  |  |  |  | 28 | 6 | 10 |
| Piperacillin-Tazobactam | 26 | 27 | 26 | 26 | 26 | 10 | 9 | 9 | 28 | 6 | 6 | 6 | 29 | 8 | 8 |
| Ticarcillin-Clavulanic Acid |  |  |  |  |  |  |  |  | 26 | 8 | 7 | 7 |  |  |  |
| Cefoperazone-Sulbactam | 25 | 24 | 24 | 23 | 25 | 7 | 18 | 8 | 26 | 6 | 7 | 8 | 23 | 6 | 8 |

**Table S5. Antimicrobial susceptibility of Gram‑positive bacteria used in this study.**

|  | ***S. aureus* ATCC 29213** | ***S. aureus* R1** | ***S. aureus* R2** | ***S. aureus* R3** | ***S. epidermidis* R1** | ***S. epidermidis* R2** | ***S. epidermidis* R3** |
| --- | --- | --- | --- | --- | --- | --- | --- |
| Gentamicin | S | S | S | S | I | I | S |
| Tetracycline | S | S | S | S | S | S | S |
| Ciprofloxacin | S | S | S | I | R | R | S |
| Trimethoprim-Sulfamethoxazole | S | S | S | S | R | R | S |
| Penicillin | R | R | R | R | R | R | R |
| Oxacillin | S | R | R | R | R | R | R |
| Vancomycin | S | S | S | S | S | S | S |
| Levofloxacin | S | S | S | S | R | R | S |
| Clindamycin | S | R | R | R | S | S | S |
| Rifampicin | S | S | S | S | S | S | S |
| Moxifloxacin | S | S | S | S | R | R | S |
| Linezolid | S | S | S | S | S | S | S |
| Tetracycline | S | S | S | S |  |  |  |
| Erythromycin | S | R | R | R | S | R | R |
| Inducible clindamycin resistance (D-test) | Negative | Positive | Negative | Negative | Negative | Positive | Negative |
| Cefoxitin screen | Negative | Positive | Positive | Positive | Positive | Positive | Positive |

S = susceptible; I = intermediate; R = resistant; ND = not determined.

**Table S6. MICs (µg/mL) of OT‑CATH against Gram‑ positive bacteria.**

|  | ***S. aureus* ATCC 29213** | ***S. aureus* R1** | ***S. aureus* R2** | ***S. aureus* R3** | ***S. epidermidis* R1** | ***S. epidermidis* R2** | ***S. epidermidis* R3** |
| --- | --- | --- | --- | --- | --- | --- | --- |
| OT-CATH | 75 | 75 | 75 | 75 | >100 | >100 | 75 |
| LL-37 | >100 | >100 | >100 | >100 | >100 | >100 | >100 |
| Gentamicin | 1.17 | 0.29 | 0.29 | 0.29 | 9.38 | 9.38 | 0.29 |
| Tetracycline | 1.17 | 0.29 | 0.59 | 0.59 | 0.59 | 0.59 | 0.59 |
| Ciprofloxacin | 0.15 | 0.29 | 0.29 | 2.34 | 37.5 | 37.5 | 0.15 |
| Trimethoprim-Sulfamethoxazole | <=1/19 | <=1/19 | <=1/19 | <=1/19 | >=4/76 | >=4/76 | <=1/19 |
| Penicillin | 37.5 | 75 | 37.5 | 75 | 37.5 | 37.5 | 37.5 |
| Oxacillin | 0.59 | 37.5 | 37.5 | 75 | 9.38 | 37.5 | 37.5 |
| Vancomycin | 0.15 | 1.17 | 1.17 | 0.29 | 1.17 | 1.17 | 1.17 |
| Levofloxacin | 0.07 | 0.07 | 0.07 | 0.59 | 18.75 | 37.5 | 0.07 |
| Clindamycin | 0.29 | 75 | 75 | 37.5 | 0.29 | 0.29 | 0.15 |
| Rifampicin | 0.29 | 0.29 | 0.29 | 0.29 | 0.29 | 0.29 | 0.3 |
| Moxifloxacin | 0.15 | 0.15 | 0.15 | 0.15 | 37.5 | 75 | 0.15 |
| Linezolid | 1.17 | 2.34 | 2.34 | 2.34 | 1.17 | 1.17 | 0.59 |
| Tetracycline | 0.07 | 0.07 | 0.07 | 0.07 |  |  |  |
| Erythromycin | 0.3 | 18.75 | 18.75 | 18.75 | 0.29 | 75 | 37.5 |

**Table S7. Zone of inhibition (mm) of OT‑CATH against Gram‑ positive bacteria determined.**

|  | ***S. aureus* ATCC 29213** | ***S. aureus* R1** | ***S. aureus* R2** | ***S. aureus* R3** | ***S. epidermidis* R1** | ***S. epidermidis* R2** | ***S. epidermidis* R3** |
| --- | --- | --- | --- | --- | --- | --- | --- |
| OT-CATH | 16 | 15 | 15 | 16 | 14 | 14 | 16 |
| LL-37 | 11 | 9 | 9 | 10 | 12 | 12 | 12 |
| Gentamicin | 28 | 25 | 26 | 26 | 16 | 16 | 28 |
| Tetracycline | 25 | 28 | 27 | 25 | 26 | 25 | 25 |
| Ciprofloxacin | 30 | 27 | 29 | 16 | 10 | 9 | 26 |
| Trimethoprim-Sulfamethoxazole | 32 | 30 | 32 | 32 | 9 | 7 | 28 |
| Penicillin | 7 | 8 | 7 | 6 | 6 | 7 | 7 |
| Oxacillin | 26 | 6 | 6 | 7 | 7 | 7 | 9 |
| Vancomycin | 24 | 24 | 23 | 23 | 24 | 25 | 24 |
| Levofloxacin | 30 | 29 | 30 | 28 | .8 | 9 | 28 |
| Clindamycin | 26 | 12 | 9 | 11 | 26 | 27 | 26 |
| Rifampicin | 29 | 26 | 28 | 27 | 29 | 29 | 29 |
| Moxifloxacin | .27 | 27 | 28 | 27 | 8 | 8 | 28 |
| Linezolid | 25 | 26 | 24 | 26 | 26 | 25 | 27 |
| Tetracycline | 26 | 27 | 24 | 26 |  |  |  |
| Erythromycin | 26 | 8 | 8 | 7 | 27 | 9 | 9 |

**Table S8. Evaluation of the MTD of OT-CATH**

| **Dose (mg/kg)** | **Dead/Survived** |
| --- | --- |
| 80 | 0/6 |
| 100 | 0/6 |
| 120 | 0/6 |
| 140 | 2/6 |

**Fig. S1. FIC indices of OT‑CATH and LL‑37 in combination with various antibiotics against ESBL‑producing *E. coli* R2.**

**Fig. S2. Representative confocal laser scanning microscopy images of *A. baumannii* R2 stained with SYTO‑9 (green) and propidium iodide (PI, red) after 1 h of treatment with 5 × MIC of the indicated peptides.** Single SYTO‑9, single PI, and merged fluorescence images are shown. Scale bar, 20 μm.

**Fig. S3. Quantification of the red-to-green fluorescence intensity ratio from SYTO 9/PI dual staining using ImageJ.**

**Fig. S4. OT-CATH disrupts bacterial cell wall integrity.** Fluorescence intensity corresponds to increased HADA incorporation into newly synthesized and damaged peptidoglycan, indicative of bacterial cell wall perturbation. PBS served as the negative control, and ampicillin (AMP) as the positive control. Bacteria were incubated with varying concentrations of agents for 30 min. Following HADA probe addition, fluorescence intensity was detected (excitation: 350 nm, emission: 420 nm). Experiments were conducted in three biological replicates; data are shown as mean ± SD. Statistical analysis was performed via one-way ANOVA, and corresponding p-values are labeled. Each symbol denotes an independent replicate.

**Fig. S5. DNA gel retardation assay. Agarose gel mobility shift assay showing that OT-CATH can bind to bacterial DNA, indicating its strong nucleic acid-binding capacity.**

**Fig. S6. Schematic diagram of competitive binding of EB with OT-CATH to DNA of bacteria**. The emission spectra of EB (20 µg/mL) bound to E. coli DNA show a characteristic peak at ~605 nm. Upon addition of increasing concentrations of OT-CATH (20–120 µg/mL), a dose-dependent decrease in EB fluorescence intensity is observed. This phenomenon indicates the displacement of EB from DNA. The control (DNA alone + EB) exhibits maximal fluorescence, while higher OT-CATH concentrations progressively quench the signal, supporting OT-CATH direct DNA-binding capability. Data represent one representative experiment.

**Fig. S7. Effect of TU on the growth curve of *A. baumannii* R2 treated with OT-CATH.** The growth profiles of bacterial cultures receiving PBS, TU alone, OT-CATH alone, or the combination of OT-CATH and TU were monitored continuously by measuring OD₆₀₀ absorbance over 18 h.

**Fig. S8. Effect of NAC on the growth curve of *A. baumannii* R2 treated with OT-CATH.** The growth profiles of bacterial cultures receiving PBS, NAC alone, OT-CATH alone, or the combination of OT-CATH and NAC were monitored continuously by measuring OD₆₀₀ absorbance over 18 h.

**Fig. S9. High-performance liquid chromatography (HPLC) profile of OT-CATH.** Reverse-phase HPLC chromatogram (C18 column, 0.1% TFA/acetonitrile gradient, detection at 220 nm) shows a dominant peak at 10.4 min corresponding to the target peptide OT-CATH-RS-40. Quantitative integration indicates this main component constitutes 95.9% of the total peak area, confirming high chemical purity. Minor peaks at 10.0 min and 10.4 min (co-eluting shoulder) represent trace impurities (<4.1% combined). The single symmetric elution profile supports successful synthesis and suitability for downstream biological assays.

**Fig. S10. Mass spectrometry (MS) analysis profile of OT-CATH.** The observed multiply charged ions ([M+nH]ⁿ⁺, n=4–9) correspond to a molecular weight of 4863.68 Da, matching the theoretical mass of OT-CATH-RS-40, confirming its correct synthesis and high purity.
